# The effects of cryopreservation on PBMCs transcriptome profile

**DOI:** 10.3389/fimmu.2025.1690316

**Published:** 2025-10-17

**Authors:** Xuan Li, Qian Chen, Jeanette Higgins, Kyndal Cook, Michael W. Baseler, Tomozumi Imamichi, Weizhong Chang

**Affiliations:** ^1^ Laboratory of Human Retrovirology and Immunoinformatics, Frederick National Laboratory for Cancer Research, Frederick, MD, United States; ^2^ AIDS Monitoring Laboratory, Frederick National Laboratory for Cancer Research, Frederick, MD, United States

**Keywords:** PBMC, cryopreservation, ScRNA-seq, immune cell, viability

## Abstract

Cryopreservation is a key method for long-term storage of biological specimens. The development of optimal cryopreservation and recovery conditions will minimize storage-related damage. The effect of the cryopreservation and recovery condition we used on peripheral blood mononuclear cells (PBMCs) has previously been evaluated using microarray analysis. The emerging single-cell RNA sequencing (scRNA-seq) technology enables deeper exploration of cellular heterogeneity and function. In the current study, we further optimized the cryopreservation and recovery procedure based on cell viability of PBMC cryopreserved for one-year evaluated using trypan blue staining and propidium iodide (PI) staining with FACS. The procedure was further validated by scRNA-seq using PBMC cryopreserved for two different lengths of time: six and 12 months, in comparison with fresh cells. We identified six major immune cell types from both fresh and recovered cryopreserved PBMCs, including monocytes, dendritic cells (DCs), natural killer (NK) cells, CD4+ T cells, CD8+ T cells, and B cells. The cell viability of all identified immune cell types was relatively stable after both six and 12 months of cryopreservation; however, the number of cells sequenced in the scRNA-seq data declined significantly by ~32% after 12 months of cryopreservation, suggesting reduced scRNA-seq cell capture efficiency. Furthermore, the transcriptome profiles of cryopreserved samples did not show substantial perturbation over the 12-month testing period, although a few key genes involved in AP-1 complex, stress response or response to calcium ion exhibited significant change, but with very small scale (< two folds). In conclusion, even though we observed a reduction of scRNA-seq cell capture efficiency after 12-month cryopreservation, our results demonstrated that the optimized cryopreservation and recovery procedure has minimal effect on PBMC viability, population composition and transcriptomic profiles after 6 or 12 months of storage.

## 1 Introduction

Cryopreservation is a process that preserves biological samples at cryogenic temperatures, maintaining the fine structure of cells for long periods of time. This storage method is broadly used for biospecimens in both basic and clinical/translational research. The procedure involves multiple steps, typically including adding a cryoprotective agent (CPA), slow freezing or vitrification, and thawing, which was thoroughly reviewed (1). Cells and tissues are typically stored at cryogenic temperatures (−196°C). At this temperature, any biological activity is practically halted, while the structural integrity of cells remains stable (2, 3). The fact that cell integrity and functionality can be effectively maintained makes cryopreserved biological samples a valuable resource for both basic research and clinical applications.

Peripheral blood mononuclear cells (PBMCs), which primarily consist of lymphocytes (B cells and T cells), are critical components of the immune system for mediating immune response during infections, vaccinations, or tumorigenesis (4). PBMCs are therefore an important resource for *in vitro* evaluation of these lymphocytes (5). In addition, monocytes within PBMCs can be induced to differentiate into macrophages (6–10) and dendritic cells (DCs) (6, 11–13) for *in vitro* studies. However, it is challenging to collect all PBMCs at the same time and study them in a fresh state. Furthermore, PBMCs from patients have also been important clinical study subjects. For example, diversity of HIV in the PBMCs of People living with HIV (PLWH) have been investigated in our group and collaborators (14–16). In these studies, cells are recovered from long-term stored PBMCs. It is necessary to recover the entire cell population from the stored PBMCs. If the population in the PBMCs were changed, the resulting diversity would be altered in the analysis and interpretation. Therefore, it is crucial to develop a method for recovering cells from long-term cryopreservation without changing the cell population and gene expression. The impact of cryopreservation on human lymphocyte functionality and phenotyping remains unclear. During the recovery phase, some cells undergo the process of cell death, altering the composition of PBMC populations. Some studies have reported significant phenotypic changes in frozen versus fresh samples, such as the impairment of cell proliferation in response to HIV-antigens, change of cytokine secretion response (17), and alteration of proportions of immune cell subsets (18, 19), as well as storage term effects on viability (20, 21). In contrast, other studies have found minimal or no impact of cryopreservation on immunophenotyping and functionality in PBMCs (22–25). Our previous work demonstrated significant changes to gene expression profiles of cryopreserved PBMCs (26); however, that study examined PBMC expression profiles as a whole PBMCs, limiting the ability to detect differences and variation between specific immune cell types. In this study, we further optimized the recovery conditions for long-term cryopreserved PBMCs. We then validated the procedure by evaluating the effects of the cryopreservation and the optimized recovery procedure on individual PBMC cell types using single-cell RNA sequencing (scRNA-seq), focused on the cell population and gene expression.

## 2 Materials and methods

### 2.1 Ethics statement

Approval for this study, including all sample materials and protocols, was granted by the National Institute of Allergy and Infectious Diseases (NIAID) Institutional Review Board, and participants were provided the informed written consent prior to blood being drawn. All experimental procedures in these studies were approved by the National Cancer Institute at Frederick and Frederick National Laboratory for Cancer Research (the protocol code number: 16–19 A6/11-30, approval date: 28 February 2021).

### 2.2 Isolation of PBMCs

PBMCs were isolated from healthy donors’ apheresis as previously described (27). Briefly, 30 mL of leukocyte suspension was laid onto 10 mL of Lymphocyte Separation Medium (MP Biomedicals, Fountain Pkwy, Solon, OH, USA) and centrifuged at 700 x g for 30 min at room temperature with the brake off. The PBMC layers were collected and then washed with PBS (Quality Biological Inc, Gaithersburg, MD, USA) three times at 500 x g for 5 min at room temperature. Cell viability was measured using trypan blue exclusion assay using Trypan Blue Stain (28) (Thermo Fisher Scientific, Waltham, MA, USA) in a 2-Chip Hemocytometer (Bulldog-Bio, Portsmouth, NH USA).

### 2.3 Freezing and thawing of PBMCs

The final optimized procedure we used is as follows: PBMCs (1,000 x 10^6^ cells) were resuspended in 10 mL of the Recovery cell Culture Freezing Medium (Gibco, Thermo Fisher Scientific), and then 1 mL of the cell suspension (100 x10^6^ cells/mL) was put into a CryoELITE cryogenic vial (Wheaton, DWK Life Science, Millville, NJ, USA) and then frozen using the CryoMed Freezer (Thermo Fisher Scientific) using freezing cycles at 1.0°C/m to -4°C, 25.0°C/m to -40°C, 10.0°C/m to -12.0°C, 1.0°C/m to -40°C, 10.0°C/m to -90°C. The frozen cells were stored in liquid nitrogen tank at -161°C until use. For FACS or scRNA-seq analysis, frozen cells were removed from storage and thawed in a 37°C water bath until a small portion of ice remained. When some portion of ice is still present in the vial, the vial was removed from the water bath and the cell suspension was then gently transferred into a 15 mL tube containing 10 mL of prewarmed RP10 medium: RPMI1640 (Thermo Fisher Scientific) supplemented with 10% heat-inactivated Fetal bovine serum (FBS) (HyClone, GE-Health Care, Chicago, IL, USA), 10 mM 4-(2-hydroxyethyl)-1-piperazineethanesulfonic acid (HEPES) (Quality Biological Inc., Gaithersburg, MD, USA) and 0.1 mg/mL of Gentamycin (Thermo Fisher Scientific). The cells were gently mixed by pipetting up and down 2–3 times, followed by centrifugation at 500 x g for 5 min at room temperature. After removing the supernatant, the cell pellet was broken up by tapping the tube gently and resuspended in 10 mL of warmed RP10. The cells were washed twice at 500 x g for 5 min at room temperature and then used in downstream assays. This thawing and recovery procedure was established through optimization with different temperatures (37°C vs 4°C), washing buffers (RP10, RP: plane RPMI-1640 or PBS) and vertexing conditions (with or without), and evaluated based on cell viability, measured with trypan blue staining and propidium iodide (PI) staining (29) with FACS, as well as cell population composition using FACS analysis.

### 2.4 FACS analysis

Fresh PBMCs or thawed cryopreserved PBMCs (2 x10^6^ cells) were washed and resuspended in 1 mL of PBS. Subsequently, 1 mL of the reconstituted Invitrogen Live/Dead Fixable Violet Dead Cell Stain Kit, for 405 nm excitation (ThermoFisher, cat # L34955) was added to the cells. Cells were mixed and incubated on ice while protected from light for 30 minutes. After incubation, cells were washed two times with cold 2% BD Pharmingen Stain Buffer (BSA) wash medium (BD Biosciences, cat # 554657, San Jose, CA), and then resuspended in 200 uL BSA wash medium. The cells were blocked with 10 μL FC-blocking medium (BD Biosciences, cat # 564219) for 10 minutes at room temperature. After blocking, 100 mL of PBMC cells were stained with 20 µL BD Multitest 6C TBNK reagent (BD Biosciences, cat#662967) for 20 minutes at 4°C. BD Multitest 6C TBNK reagent consists of CD3-Fluorescein isothiocyanate (FITC), CD16/56- R-Phycoerythrin (PE), CD45- Peridinin-chlorophyll proteins (PerCP-Cy5.5), CD4-PE-Cyanine7 (PE-Cy7), CD19 Allophycocyanin (APC) and CD8-Allophycocyanin Cyanine 7 (APC-Cy7) monoclonal antibodies. Afterward, the cells were lysed with 450 μL 1X BD FACS Lysing solution (BD Biosciences, cat# 349202), washed twice in cold 2% BSA wash medium, and resuspended in 500 µL 2% cold BSA wash medium. Samples were analyzed immediately on a Becton Dickinson FacsLyric flow cytometer (BD Biosciences). Veri-cells (Biolegend, cat # 452002, San Diego, CA) were used as controls for the assay. Lymphocytes were enumerated using CD45 PerCP versus side scatter (SSC) gating. Dead cells were excluded using the Invitrogen Live/Dead Fixable Violet Dead Cell Stain Kit, for 405 nm excitation (ThermoFisher, cat # L34955). A minimum of 5,000 lymphocytes were collected for each sample and analyzed with BD FacsSuite software (BD Biosciences). Flow cytometric analysis provided quantification of the proportions of CD3+ T cells, CD4+ T cells, CD8+ T cells, CD19+ B cells, and CD16 + 56+ natural killer (NK) cells.

### 2.5 Construction of scRNA-seq libraries

Cells from three independent donors were washed three times with RP10 media, and cell viability and cell counts were determined using a Cellometer Auto 2000 (Nexcelom Bioscience, Lawrence, MA, USA) with ViaStain AOPI Staining (30) Solution (Nexcelom Bioscience). Consistently, the cell viability was 98% to 100%. Each sample was resuspended at 1,000 cells/µL in RP10. The scRNA-seq libraries were constructed using the Chromium Next GEM Single Cell 5′ Reagent Kit V2 (Dual Index) (10x Genomics, Pleasanton, CA, USA) following the manufacturer’s instructions. Briefly, a targeted recovery of 10,000 cell suspension with the master mix containing reverse transcription (RT) reagent and template switch oligos and RT enzyme was loaded into a Chromium Chip K (10x Genomics). The single-cell GEM generation and barcoding followed by cDNA synthesis were run on the Chromium Chip K in the 10x Chromium Controller (10x Genomics). The cDNA was amplified by 13 PCR cycles. Based on Agilent 2100 Bioanalyzer (Agilent Biotechnology, Santa Clara, CA, USA) and Qubit 4 analysis (Thermo Fisher Scientific), the scRNA-seq libraries contained a single peak of DNA between 350 and 500 bp (average fragment sizes were 450–550 bp).

### 2.6 Data curation, processing, and analysis

Sequence data were initially processed and quality evaluated with the Cell Ranger pipeline (v7.0.1, 10x Genomics, Pleasanton, CA, USA) using human reference GRCh38-2020-A (10x Genomics). Cells with more than 600 detected genes and less than 10% of reads mapped to the mitochondrial genome were kept for downstream analysis. The scRNA-seq data analysis was conducted with the Seurat package (v5.0.1) (31). Samples were integrated and batch effects were corrected by the Harmony method in Seurat. Primary cell types were identified by the MapQuery method in Seurat using a reference human PBMC (32) and confirmed by the expression of known cell marker genes. Doublet prediction was carried out with the SCDdFinder package (v.1.23.4) and cells predicted to be doublets were removed from the analysis. Differentially expressed genes (DEGs) were determined at the single-cell level for each individual donor using Seurat’s FindMarkers function with Wilcoxon Rank Sum test (default setting), with a set of very low thresholds: minimum log2 fold change of 0.1 and an adjusted p-value of less than 0.05. Pseudobulk differential expression analysis was carried out with the DESeq2 package (v1.46.0) (33). The Wald test was used to identify genes that are differentially expressed by pairwise comparison between different cryopreservation time points (6 or 12 months) versus fresh samples. Multiple test correction was implemented using the Benjamini-Hochberg false discovery rate (FDR). Significant genes were identified as showing at least 1.5-fold change with a BH adjusted Wald test *p*-value less than 0.05 (*p.adj* < 0.05). DEG list functional annotation was determined with DAVID (34).

## 3 Results

### 3.1 Establishment of cell recovery

**Figure 1:**
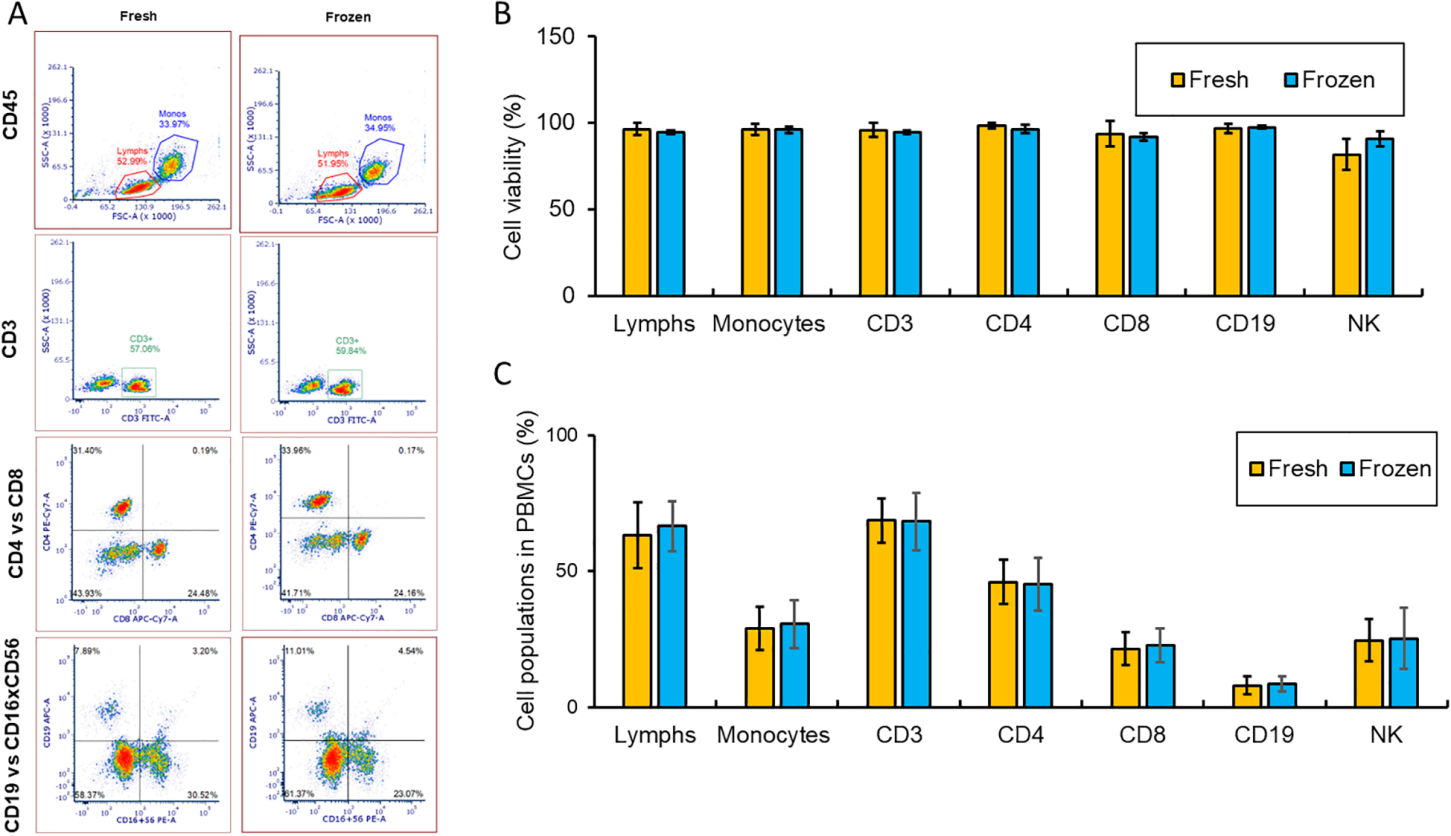
The cell populations in fresh and 12 months frozen PBMCs were analyzed using FACS analysis using 10 independent donors. **(A)** One of the representative results from FACS is shown. CD45 staining was used to distinguish monocytes and lymphocytes. CD3 is a marker of T cells, CD19 and CD16xCD56 are markers of B and NK cells, respectively. **(B, C)** Comparison of each cell population and viability in fresh and recovered after 12 months storage from 10 different donors are summarized. Results show mean ± SD.

Several methods were compared to establish the most effective protocol for recovering cells from frozen PBMCs based on viability using trypan blue exclusion assay. The tested conditions involved altering the temperature of the medium at 4°C or 37°C for washing thawed cells, using different buffers, and with or without vertexing. Washing cells with the prewarmed at 37°C RP10 medium without vertexing proved to be the most effective method for the recovery of the largest number of cells from PBMCs cryopreserved for 12 months, as assessed by trypan blue staining (**Table 1**). The composition of recovered cell populations was first measured by FACS analysis from PBMCs of 10 independent donors (**Figures 1A**). There were no statistically significant differences in the proportions of monocytes, lymphocytes, CD3+, CD4+, CD8+, CD19+ and NK cells between fresh and 12-month-frozen PBMCs, as determined by PI staining and FACS (**Figures 1B, C**).

**Table 1:** Optimization of the recovering condition.

Effect of temperature of wash buffer											
Tube No	Cell No (M*)	1st wash	Temp.	2nd wash	Temp.	3 wash	Temp.	Live cell No	Dead Cell	Live Cell (M*)	Viability %
1	5	RP10	37	RP10	37	RP10	37	119	1	4.76	95.20%
2	5	RP10	4	RP10	4	RP10	4	83.5	2	3.34	66.80%
3	5	PBS	37	PBS	37	PBS	37	93	4	3.72	74.40%
4	5	PBS	4	PBS	4	PBS	4	64	6.5	2.56	51.20%

Wash buffer effect: RP10, RP and PBS with washing at 37°C											
Tube No	Cell No (M*)	1st wash	Temp	2nd wash	Temp	3rd wash	Temp.	Live cell No	Dead Cell	Live cell (M*)	Viability %
5	5	RP10	37	RP10	37	RP10	37	120	1.5	4.8	96.00
6	5	RP	37	RP	37	RP	37	89.5	4	3.58	71.60
7	5	PBS	37	PBS	37	PBS	37	85	7	3.40	68.00

Effect of vortex									
Tube No	Cell No (M*)	1st wash	2nd wash	3rd wash	Vortex	Live cell No	Dead Cell	Live Cell (M*)	Viability%
8	5	RP10	RP10	RP10		95	1	4.75	95
9	5	RP10	RP10	RP10	yes	86	1	4.3	86
10	5	PBS	PBS	PBS		75	10	3.75	75
11	5	PBS	PBS	PBS	Yes	63	11	3.15	63

*M, x 10^6^ cells; Rows with red font were the best condition for each set of tests. Top row in each section was the best condition tested.

**Figure 2:**
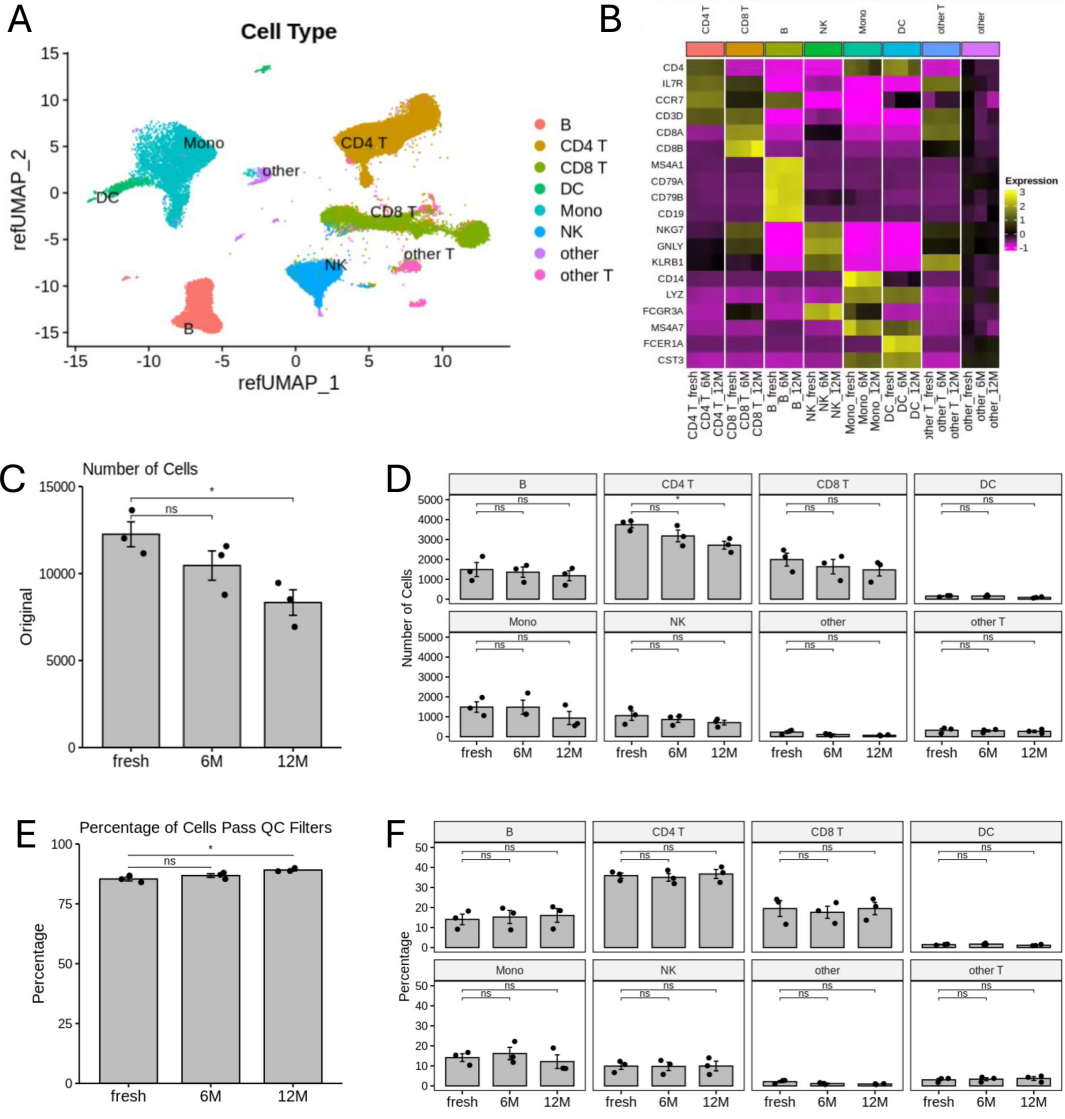
Identification of the major immune cell types of PBMCs and determination of the effect of the cryopreservation and recovery procedure. **(A)** UMAP plot showing the clusters of primary immune cell types identified in PBMCs. **(B)** Heatmap plot representing the expression of marker genes for each immune cell type (CD4+ T: CD4, IL7R, CCR7, CD3D; CD8+ T: CD8A, CD8B; B: MS4A1, CD79A, CD79B, CD19; NK: NKG7, GNLY, KLRB1; Monocytes: CD14, LYZ, FCGR3A, MS4A7; DC: FCER1A, CST3). **(C)** Bar plot representing the number of sequenced cells for fresh, 6-month or 12-month cryopreserved samples. **(D)** Bar plots representing the number of cells passed QC filters from the sequenced cells of each cell type for fresh, 6-month or 12-month cryopreserved samples. **(E)** Bar plot representing percentages of cells passing QC filters from total sequenced cells for fresh, 6-month or 12-month cryopreserved samples. **(F)** Bar plots representing percentages of each cell type from total cells passing QC filters for fresh, 6-month and 12-month cryopreserved samples. In **(C–F)**: Each dot represents a sample, *: p < 0.05 and ns, non-significant.

The data quality was consistent for all nine samples from scRNA-seq analysis with detailed quality control metrics summarized in **Supplementary Table 1**. The number of UMI per cell are very consistent with the media from 3235 to 4441 (**Supplementary Figure S1A**). Cells with more than 600 detected genes (**Supplementary Figure S1B**) and less than 10% of reads mapped to the mitochondrial genome (**Supplementary Figure S1C**) were kept for downstream analysis. The subsequent scRNA-seq analysis identified six major immune cell clusters including monocytes, DCs, NK cells, CD4+ T cells, CD8+ T cells, and B cells, as well as two small clusters labeled as “other” for the clusters which mostly represent non-immune cells (e.g. Platelets, Erythrocytes, stem cells, etc.), and “other T cells” for the clusters that include any T cells other than CD4^+^ and CD8^+^ T cells. (**Figure 2A**). For downstream analysis, we focused on these six major immune cell types. These immune cell types were confirmed by expression of their respective cell-type specific marker genes (**Figure 2B**). The number of sequenced PBMCs of the 12-month cryopreserved samples decreased significantly by about 32% compared with the fresh PBMCs, while the number of sequenced cells decreased 15% but not significantly after 6 months of cryopreserve-tion (**Figure 2C**, **Supplementary Figure S2A**). After filtering low-quality cells (<600 detected genes, or >10% of reads mapped to the mitochondrial genes), the number of remaining high-quality cells retained for analysis showed the same pattern of decline: a significant 29% decrease in cell number for PBMCs cryopreserved for 12 months compared with the fresh group and a non-significant 13% decrease for PBMCs cryopreserved for 6 months (**Supplementary Figure S2B**, **S2C**). Consistently, the number of high-quality cells per immune cell type within the sequencing data also declined in cryopreserved PBMCs, but a significant decrease was only observed in CD4^+^ T cells in PBMCs cryopreserved for 12 months (**Figure 2D**, **Supplementary Figure S2D**). Compared with fresh PBMCs, the proportion of high-quality cells relative to all sequenced cells was slightly increased for PBMCs cryopreserved for 6 months (1.64%) or 12 months (4.33%, *p* = 0.025) (**Figure 2E**). The pattern consistently reflected the change for each sample at each time point for each donor (**Supplementary Figure S2E**). This is consistent with other *in-vitro* viability assessments of human primary cells (25), indicating that the optimized cryopreservation and recovery procedure can maintain reasonable PBMC viability for at least 12 months of storage. Furthermore, the percentage of high-quality cells for each immune cell type remained relatively stable at both 6-month and 12-month time points for all the donors combined (**Figure 2F**) and for each donor individually (**Supplementary Figure S2F**), suggesting that no specific immune cell type was disproportionally affected by cryopreservation using the optimized procedure.

### 3.2 Analysis of differentially expressed genes

**Figure 3:**
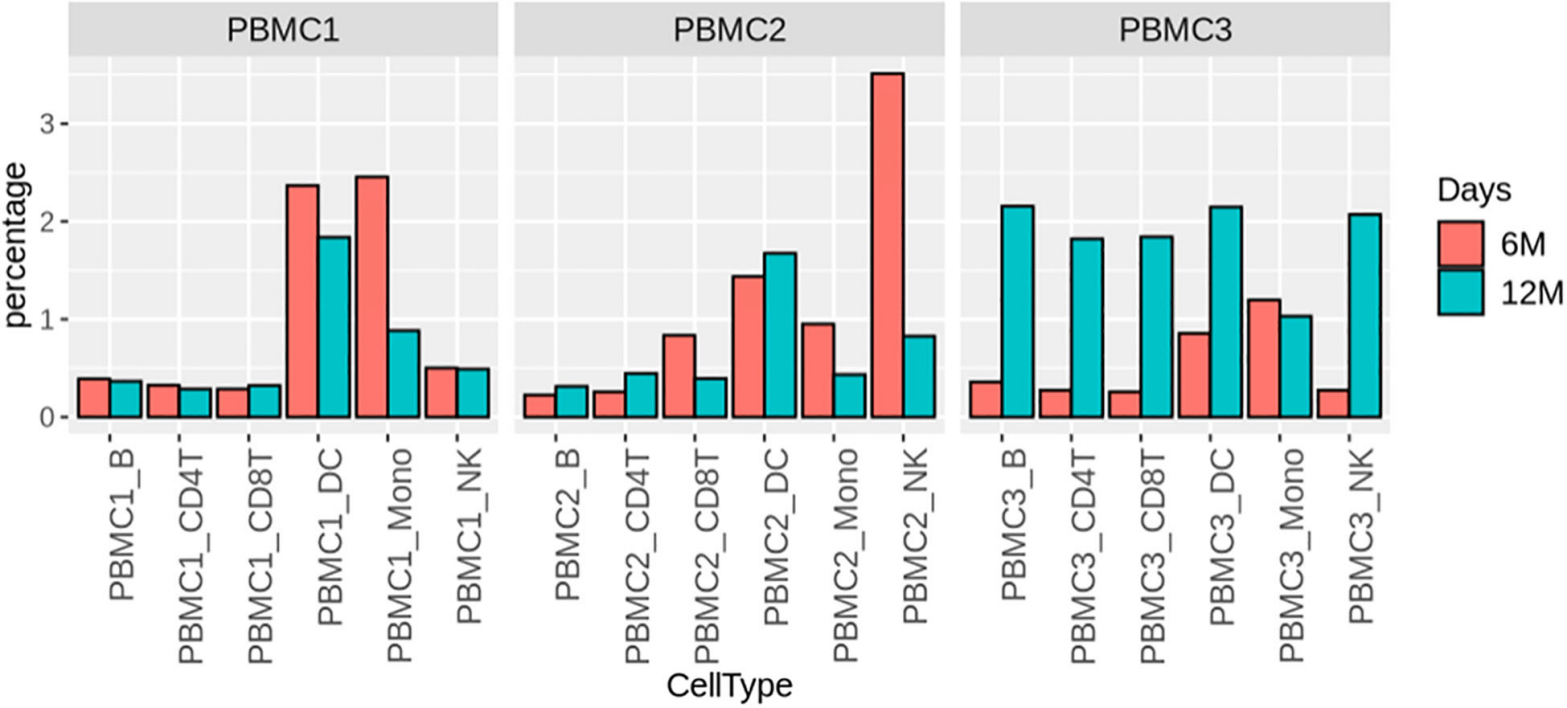
Summary of differential gene expression gene analysis for major cell types. Bar plots depicting the percentage of differentially regulated genes in each immune cell type after being cryopreserved for 6 (red) and 12 (cyan) months vs fresh.

To investigate the influence of cryopreservation duration on gene expression profiles, we first performed pseudobulk differential expression analysis on cryopreserved PBMCs (6 or 12 months) vs. fresh PBMCs grouped by time points, where gene counts were aggregated in each sample. This approach allows us to detect differences between conditions beyond variation among donors. For all immune cell types examined, only a few DEGs were identified: from 6 DEGs in DC cells for 6-month cryopreserved vs fresh to 50 DEGs in CD4^+^ T cells for 12-month cryopreserved vs fresh (**Supplementary Figure S3**). The detailed DEG lists are listed in **Supplementary Data 1**. The results suggest that 12 months of cryopreservation had little effects on gene expression profiles. However, this bulk analysis could potentially average out some DEGs that can occur when merging data from multiple donors. In addition, cells from each donor can also respond to cryopreservation very differently. To better understand this donor heterogeneity, we further performed differential expression analysis on cryopreserved PBMCs (6 or 12 months) vs. fresh PBMCs for each donor separately. This approach avoids the potential averaging out of some DEGs that can occur when merging data from multiple donors. For each of the six major immune cell types, all identified DEGs (both upregulated and downregulated) were visualized in separate heatmaps for the 6- and 12-month comparisons with fresh for donor 1(**Supplementary Figures S4**), donor 2 (**Supplementary Figure S5**) and donor 3 (**Supplementary Figure S6**). The lists of DEGs for each cell type for the 6- and 12-month comparisons with fresh were presented for each donor: donor 1(**Supplementary Data 2**), donor 2 (**Supplementary Data 3**) and donor 3 (**Supplementary Data 4**). The number of DEGs for each cryopreserved sample ranged from 55 to 875 (**Supplementary Table 2**, **Supplementary Figure S7**), representing 0.22 - 3.51% of the 24,916 total genes in the transcriptome (**Figure 3**), demonstrating that gene expression profiles for PBMCs were not substantially perturbed during 12-month cryopreservation. Nonetheless, some immune cell types exhibited slightly greater transcriptomic responses to cryopreservation, with more DEGs detected in monocytes, NK cells, and DCs than in CD4+ T cells, CD8+ T cells, and B cells (**Supplementary Table 2**, **Supplementary Figure S7**).

**Figure 4:**
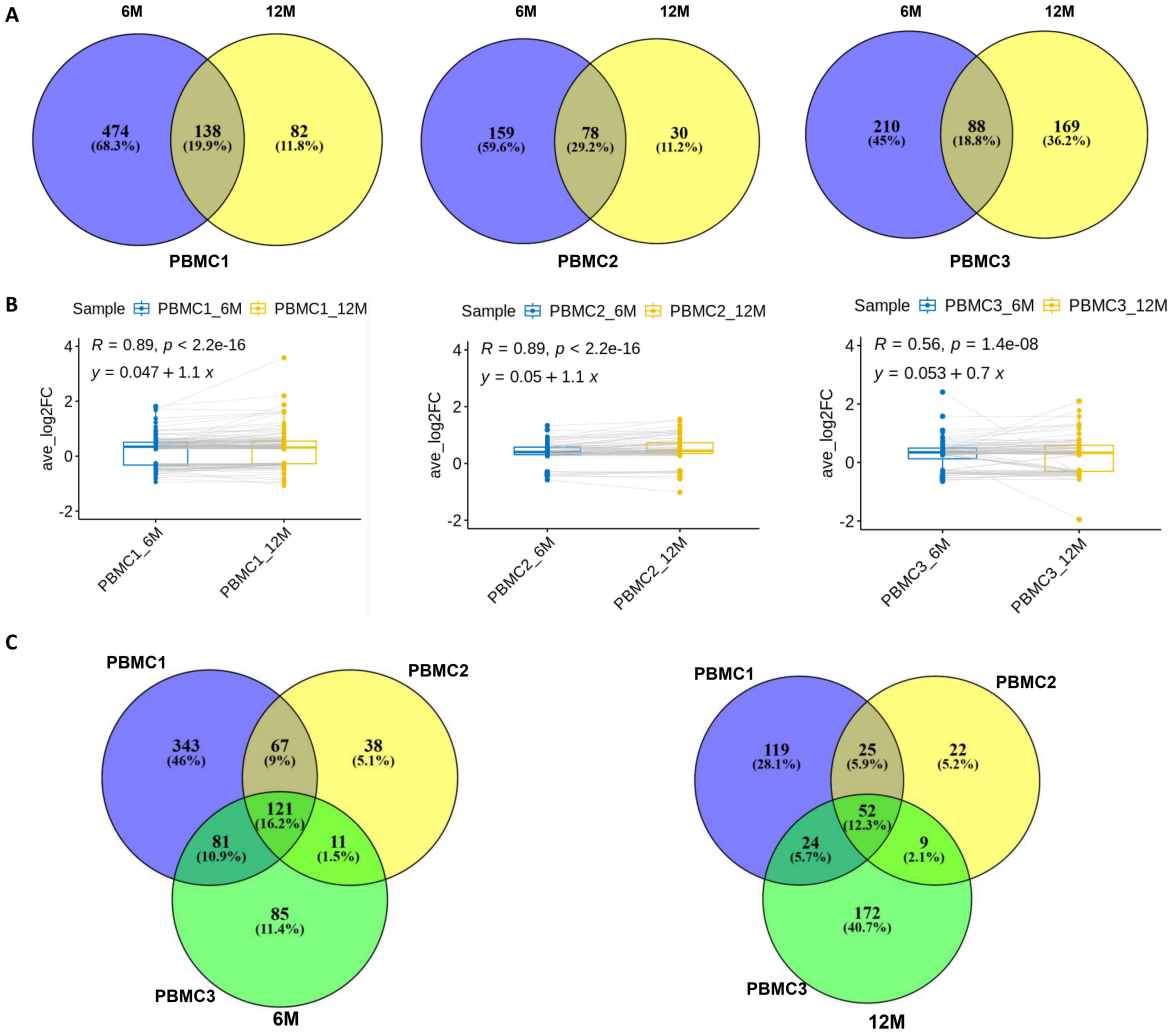
Differential expression gene (DEG) analysis of monocytes with different cryopreservation times. **(A)** Venn Diagram analysis of DEGs identified from monocytes of PBMCs cryopreserved for 6- and 12-month vs. fresh PBMCs for each donor. **(B)** Paired-boxplot of expression fold-change of common DEGs identified from monocytes of PBMCs cryopreserved for 6- and 12-month vs. fresh PBMCs for each donor. The same gene was linked by the line. **(C)** Venn Diagram analysis of DEGs identified from monocytes of PBMCs cryopreserved for 6- or 12-month vs. fresh PBMCs for all three donors (Left: 6-month vs Fresh; Right: 12-month vs fresh).

To determine whether the identified DEGs were associated with the cryopreservation and recovery, the common DEGs of each cell type between the two time points (6- and 12-month) vs. fresh were identified for each donor. Venn diagram analysis in monocytes (**Figure 4A**) revealed that most DEGs were unique to a given donor and time point with relatively few genes shared. The unique DEGs would likely be averaged out in biological replicates in the study. Despite this, Fisher’s exact test showed that the common genes were significant for all 6- and 12-month sample pairs, suggesting their potential biological relevance. For DEGs common between 6- and 12-month samples in a given donor, we explored their fold-change in expression between the time points (**Figure 4B**, **Supplementary Table 2**). The majority of these common DEGs were only mildly up- or down-regulated (within a two-fold range), and the direction and level of regulation was generally consistent between the two time points. Linear regression of fold changes between 6- and 12-month time-points yielded linear coefficients (slope) centered around 1 (Supplemental **Table 2**) and R-squared values above 0.85 for the majority of comparisons, further supporting the stability of transcriptome profiles over time. Furthermore, we extracted the common DEGs among all three donors at either 6 or 12 months respectively and conducted a Fisher’s extract test. The results demonstrated that the common DEGs between these two time-points also displayed significant overlap (**Figure 4C**, **Table 2**). Similar findings were observed in DCs (**Supplementary Figure S8**, **Supplementary Table 2**, **Table 2**) and NK cells (**Supplementary Figure S9**, **Supplementary Table 2**, **Table 2**). The same analysis was also performed for the other three cell types with less DEGs: CD4^+^ T cell, CD8^+^ T cell and B cell. The results were again consistent (**Table 2**, **Supplementary Table 2**). The details of these shared DEGs in each cell type were listed in **Supplementary Table 3**. Taken together, these observations suggest that the optimized cryopreservation and recovery procedure did not significantly influence the transcriptome of these immune cell types.

**Table 2:** Summary of shared differentially expressed genes (DEGs) for each cell types.

Cell type	Shared DEGs within 6 month	Shared DEGs within 12 month	Shared DEGs between 6- and 12-month	P-value (<)
Monocytes	121	52	36	2.2 X 10 ^-16^
Dendritic cells	40	64	17	2.2 X 10 ^-16^
NK	15	33	7	2.2 X 10 ^-16^
CD4+ T cells	20	29	14	2.2 X 10 ^-16^
CD8+ T cells	18	26	9	2.2 X 10 ^-16^
B cells	16	23	8	2.2 X 10 ^-16^

### 3.3 Functional analysis of DEGs arising from the cryopreservation and the optimized recovery procedure

**Figure 5:**
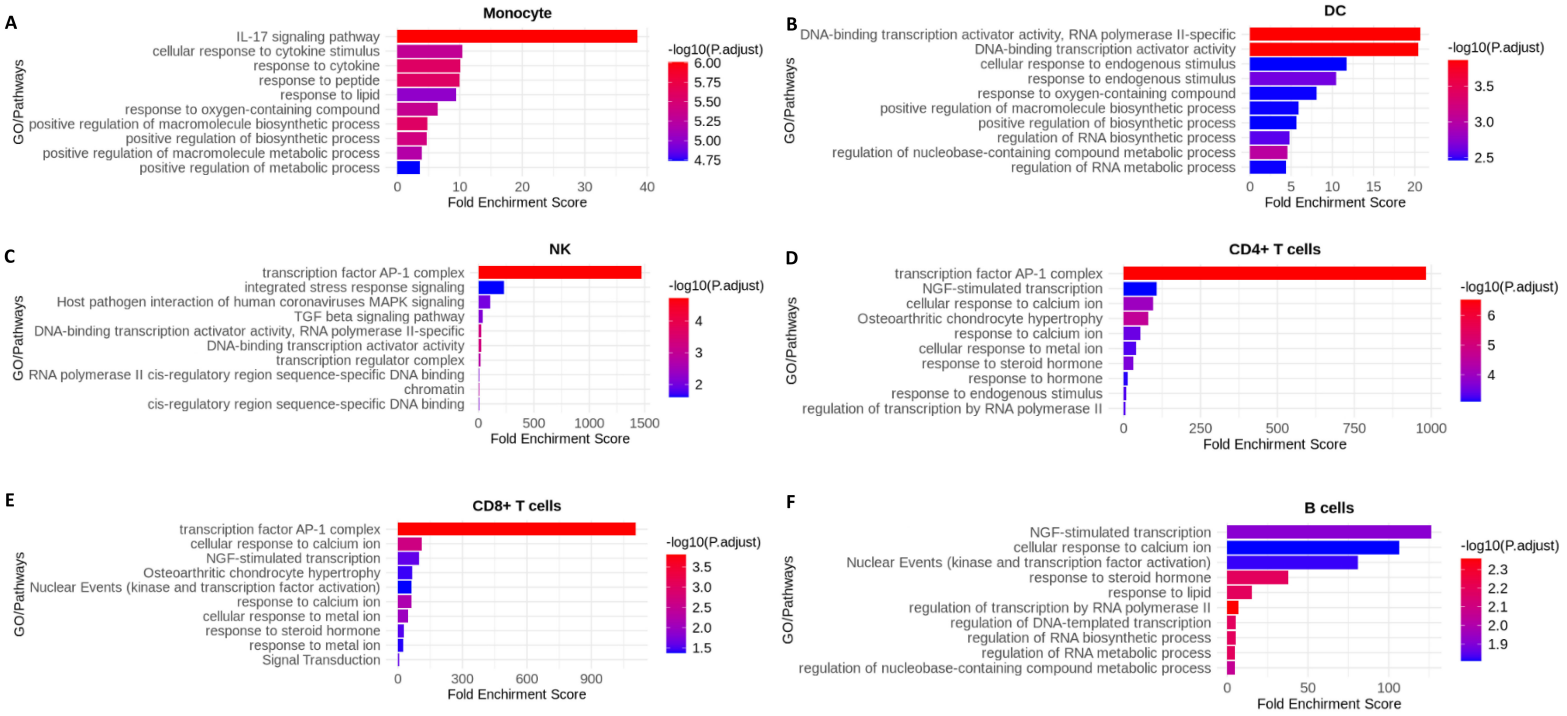
Top 10 most significantly enriched functional terms or pathways identified from the common identified from each cell type of PBMCs cryopreserved for 6- and 12-month vs. fresh PBMCs for each donor. **(A)** Monocyte; **(B)** DCs; **(C)** NK cells; **(D)** CD4+ T; **(F)** CD8+ T cells; **(E)** B cells.

Although the optimized cryopreservation and recovery procedure did not significantly affect the PBMC cell populations or transcriptomic profiles, the common genes we identified were not random and are likely related to the cellular response to the procedure. Therefore, we performed a functional annotation analysis of the common DEG lists from all six major immune cell types (**Supplementary Table 3**) using DAVID. The enriched terms or pathways for each cell type filtered by Benjamini adjusted *p* value < 0.05 and FDR <10 were listed in **Supplementary Table 4**-**9** and the top 10 most significant terms/pathways were visualized in **Figure 5**. Interestingly, the common DEGs for each cell type had distinct characteristics. Among the top 10 list, the term “transcription factor AP-1 complex” is present in three out of six cell types: NK (**Figure 5C**), CD4^+^ T cells (**Figure 5D**) and CD8+ T cells (**Figure 5E**), and it is also present in the B cells list outside of the top 10 (**Supplementary Table 9**). Moreover, the terms relating to DNA transcription, RNA biosynthetic, and metabolic processes were present in the top 10 of DCs (**Figure 5B**) and B cells (**Figure 5F**). Similar terms were also present outside of the top 10 in monocytes (**Supplementary Table 4**) and CD4^+^ T cells (**Supplementary Table 7**). Furthermore, the term “response to calcium ion” was present among the top 10 enriched terms in CD4^+^ T cells (**Figure 5D**), CD8+ T cells (**Figure 5E**) and B cells (**Figure 5F**). Finally, we observed the enriched term, “cellular response to stress”, in monocytes (**Supplementary Table 4**) and CD4^+^ T cells (**Supplementary Table 7**).

## 4 Discussion

In this study, we optimized a cryopreservation and recovery procedure capable of preserving PBMCs for up to 12 months with minimal loss of viability assessed using trypan blue staining to detect dead cells (**Table 1**). The procedure was confirmed using PI staining and FACS analysis were used to measure cell viability (detecting late-stage apoptotic and necrotic cells) and proportion of major cell types (**Figures 1B, C**). We further validated this optimized procedure by comparing the PBMCs cryopreserved for 6 or 12 months with fresh PBMCs from three independent donors using scRNA-seq. The number of sequenced cells was unaffected after 6 months of cryopreservation, but a significant decrease was observed after 12 months (**Figures 2C, D**), suggesting long storage (12 months or longer) may reduce scRNA-seq cell capture efficiency. The cause of this decline is unknown and warrants further investigation but is beyond the scope of this study. Further analysis found that the proportion of cells passed QC filters was similar for fresh, 6-month and 12-month cryopreserved PBMCs when evaluated across all cells (**Figure 2E**) and within each cell type (**Figure 2F**), suggesting that 6-month or 12-month cryopreservation with the optimized procedure has no effect on cell viability, consistent with the results evaluated with trypan blue staining (**Table 1**) and PI staining in FACS analysis (**Figures 1B, C**). Previous studies, including our own, have reported declines in cell viability after long-term cryopreservation when alternative recovery methods were used (25, 26). Our results suggest that the improved quality of PBMCs from this optimized procedure will benefit future studies and other applications requiring long-term sample storage.

Across the immune cell types we examined, all displayed a stable viability and the proportions of immune cell types in PBMCs remained stable across all donors after long-term cryopreservation. Likewise, transcriptomic profiles showed no significant change in any major PBMC immune cell type examined, demonstrating that our optimized cryopreservation and recovery procedure effectively maintains cellular functionality.

A limited number of DEGs were identified in recovered PBMCs cryopreserved for 6 or 12 months. Most of these DEGs are unique to individual donors and time points (**Figure 4**, **S8**, **S9**, **Table 2**, **Supplementary Table 2**) highlighting the phenotypic heterogeneity among biological samples which must be considered when interpretating results from biomedical experiments (35). This heterogeneity also occurs within each sample, as diversity can manifest at genomic and transcriptomic levels for individual cell. Bulk analysis often only describes the average profile of the population. Thus, it is important to perform analysis at single-cell resolution to identify cell type-specific phenotyping and transcriptomic profiles. However, the DEG number is minimal compared with the whole human genome and most of these DEG changes were less than two-fold, further demonstrating our cryopreservation and optimized recovery procedure had minimal effect on the transcriptome of PBMCs.

Functional annotation analysis using DAVID showed that the common DEGs from different cell types had different functional profiles (**Figure 5**, **Supplementary Table 4**-**9**). The term “transcription factor AP-1 complex” was identified in four out of six cell types: NK (**Figure 5 C**), CD4^+^ T cells (**Figure 5D**), CD8+ T cells (**Figure 5E**) and B cells (**Supplementary Table 9**). Among the genes in this complex, Jun family and FOS/FOSB family genes are of particular interest, as they play critical roles in regulation of cell proliferation, differentiation, and transformation (36). The terms related to DNA transcription, RNA biosynthetic, and metabolic processes were present in DCs (**Figure 5B**) and B cells (**Figure 5F**). These genes might be responsible for the stress generated by the cryopreservation and recovery process. We also observed the enriched term, “cellular response to stress”, in monocytes (**Supplementary Table 4**) and CD4^+^ T cells (**Supplementary Table 7**). Interestingly, we also found that some genes related to calcium in top 10 most enriched terms/pathways in CD4^+^ T cells (**Figure 5D**), CD8+ T cells (**Figure 5E**) and B cells (**Figure 5F**), potentially reflecting stimulation by ions in the recovery buffer. These findings are consistent with our previous study (26), which identified a larger number of DEGs (>1,300) due to differences in the cryopreservation and recovery procedures used. Multiple research groups consistently reported detecting gene expression change in both cell proliferation and cytokine expression in PBMCs (37–39). We also identified the enriched term, “response to cytokine”, in monocytes (**Figure 5A**, **Supplementary Table 4**) and DCs (**Supplementary Table 5**).

In this study, we focused on developing a procedure to recover a similar cell population and gene expression in long-term cryopreserved PBMC compared to fresh PBMC and found little effect of the cryopreservation and recovery procedure on the cell viability, cell population and gene expression comparing the PBMC stored up to 12 months with fresh PBMC. Therefore, our optimized procedure can be used in gene expression study for various PBMC samples such as investigation of diversity of HIV in the PBMCs of People living with HIV (PLWH) at the National Institute of Allergy and Infectious Diseases (NIAID) in the National Institutes of Health (Bethesda, Maryland, USA) (14–16). However, except only limited number of cell markers were used to sort cell types of PBMCs (**Figure 1A**), we haven’t evaluated antigenicity level and functional state of cryopreserved and fresh PBMCs. We haven’t compared immunological response of the cryopreserved and fresh PBMCs. The impact of cryopreservation on immunophenotype and functionality of PBMCs have been investigated by many groups. It was demonstrated that most surface antigens (CD3, CD4, CD8, CD19, CD16/56) are preserved after cryopreservation, though some activation markers show changes (2). Another report showed cryopreserved mononuclear cells preserved antigenicity and T-cell receptor recognition, with some decrease in proliferative response (40). Moreover, it had been proved that PBMCs could maintain functional responsiveness in ICS and ELISPOT assays if cryopreservation protocols are optimized (41). Furthermore, CD4+ and CD8+ cells in cryopreserved human PBMC could maintain full functionality in cytokine ELISPOT assays (24). Therefore, our optimized cryopreservation and recovery procedure needs to be further validated by evaluating the antigenicity and functional state of cryopreserved and fresh PBMCs and by performing functional assay to compare the functionality of cryopreserved and fresh PBMCs. However, this is beyond the scope of this study and is planned in the future study to broaden the use of this optimized procedure.

In conclusion, although we observed a reduction of scRNA-seq cell capture efficiency after 12-month cryopreservation, our optimized cryopreservation and recovery procedure minimally affects PBMC viability, population composition, and transcriptomic profiles after 6 or 12 months of storage.

## Data Availability

The raw sequencing data used in this study have been deposited into NCBI Gene Expression Omnibus (GEO): GSE304704. The analysis codes have been deposited to GitHub: https://github.com/LHRI-Bioinformatics/Cryo_PBMC.
