# Supplementary material for: The effects of cryopreservation on PBMCs transcriptome profile
Source: Front Immunol. 2025 Oct 17;16:1690316. doi: 10.3389/fimmu.2025.1690316 (PMC12575299; doi:10.3389/fimmu.2025.1690316)
Supplement: Supplementary file 5 [file Table1.docx]

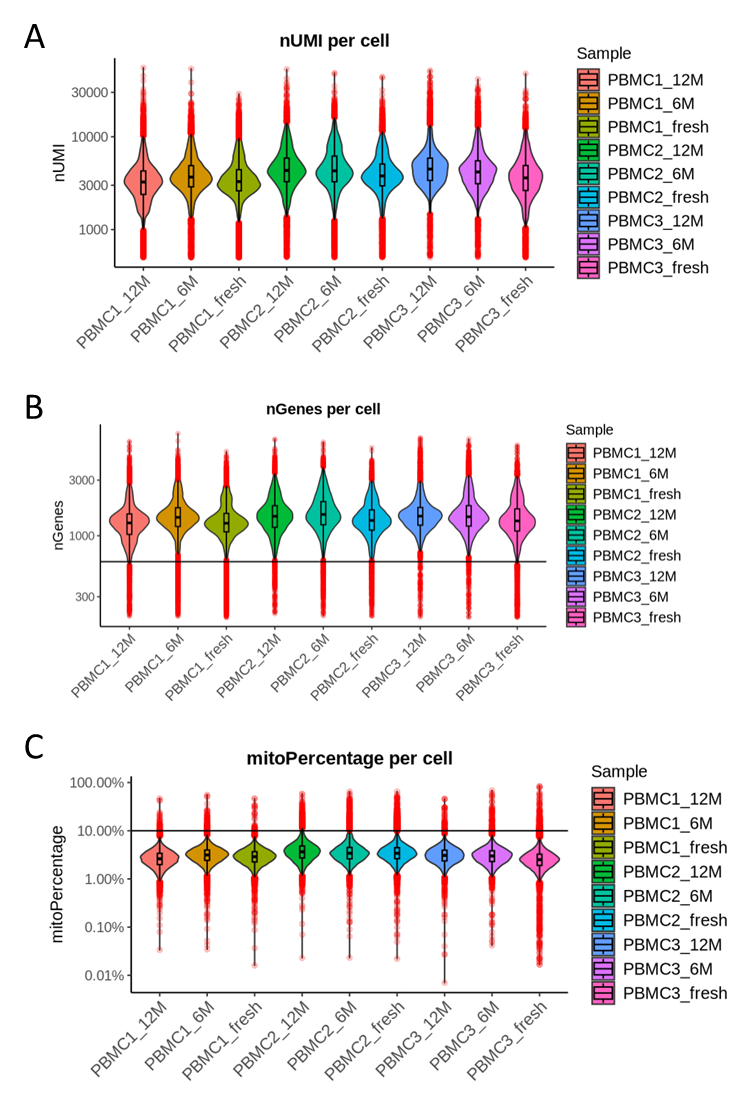
Supplementary Material

# Figure S1. Assessment of the scRNA-seq data of fresh and recovered PBMCs from cryopreservation for 6 and 12 months. (A) Number of UMI (nUMI) per cell for each sample. (B) Number of Genes (nGenes) per cell for each sample. (C) Percentage of mitochondria gene distribution: Percent of genes mapped to mitochondrial genome.

**
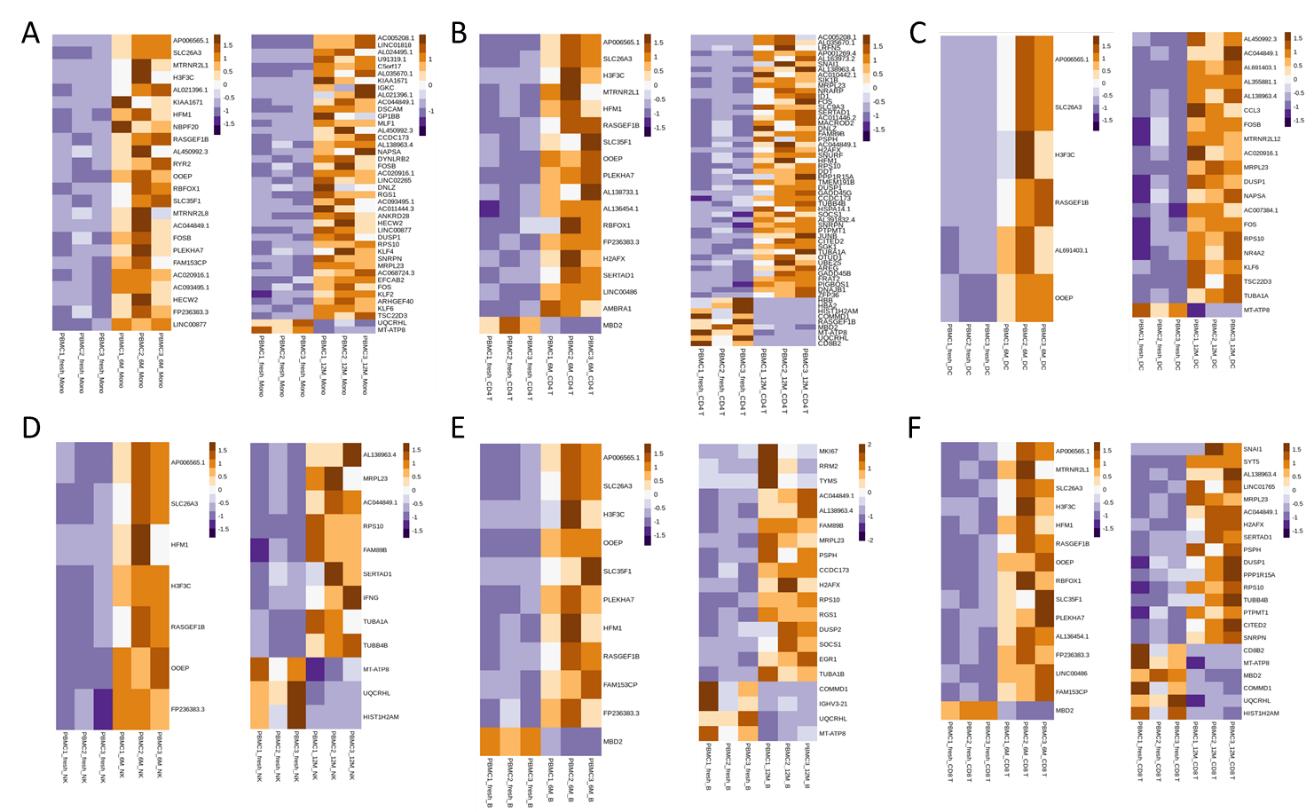
**
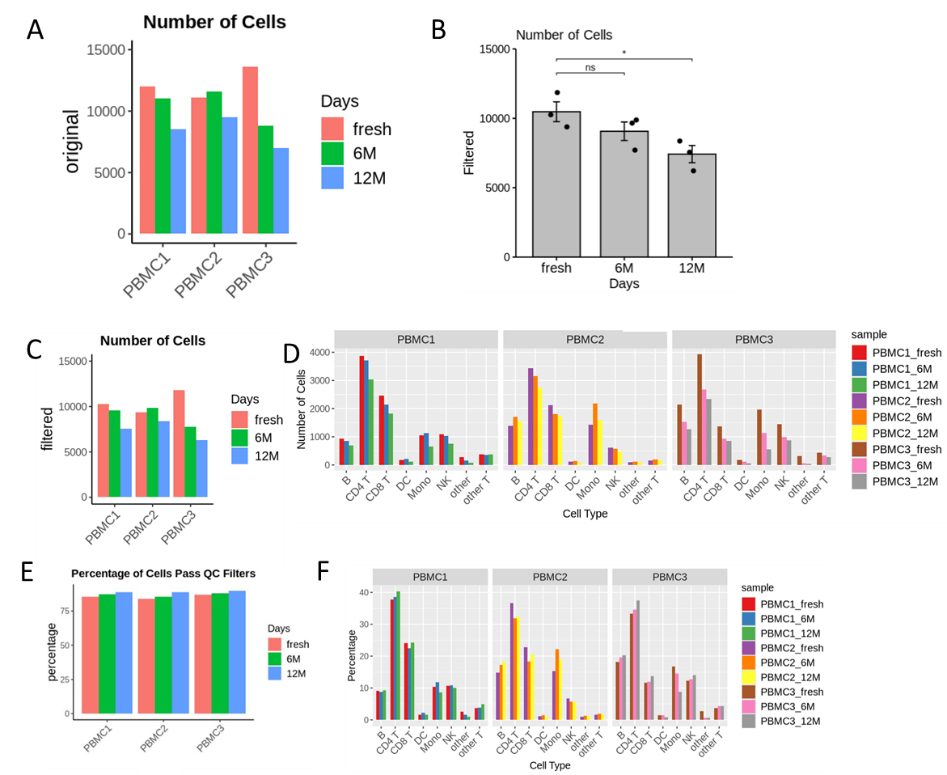
**Figure S2.** **Number of the sequenced cells from fresh PBMCs or PBMCs cryopreserved for 6 months or 12 months.** Bar plots for (A) the number of sequenced cells for each donor at the different time points; (B)the number of cells passed QC filters from sequenced cells for fresh PBMCs or PBMCs cryopreserved for 6 months and 12 months; (C) the number of cells passed QC filters from sequenced cells for each donor at the different time points; (D) the number of cells for each cell type passed QC filters from the sequenced cells for each donor at the different time points; (E) percentages of cell passed QC filter for each donor at the different time points; (F) percentage of cells passed QC filter for each cell type for each donor at the different time points. *: *p* < 0.05 and ns: non-significant.

**Figure S3. Heatmap of pseudobulk-DEGs identified from different cell types for different cryopreserved time versus fresh.** (A) Monocyte; (B) CD4+ T; (C) DCs; (D) NK cells; (E) B cells; (F) CD8+ T cells.

**
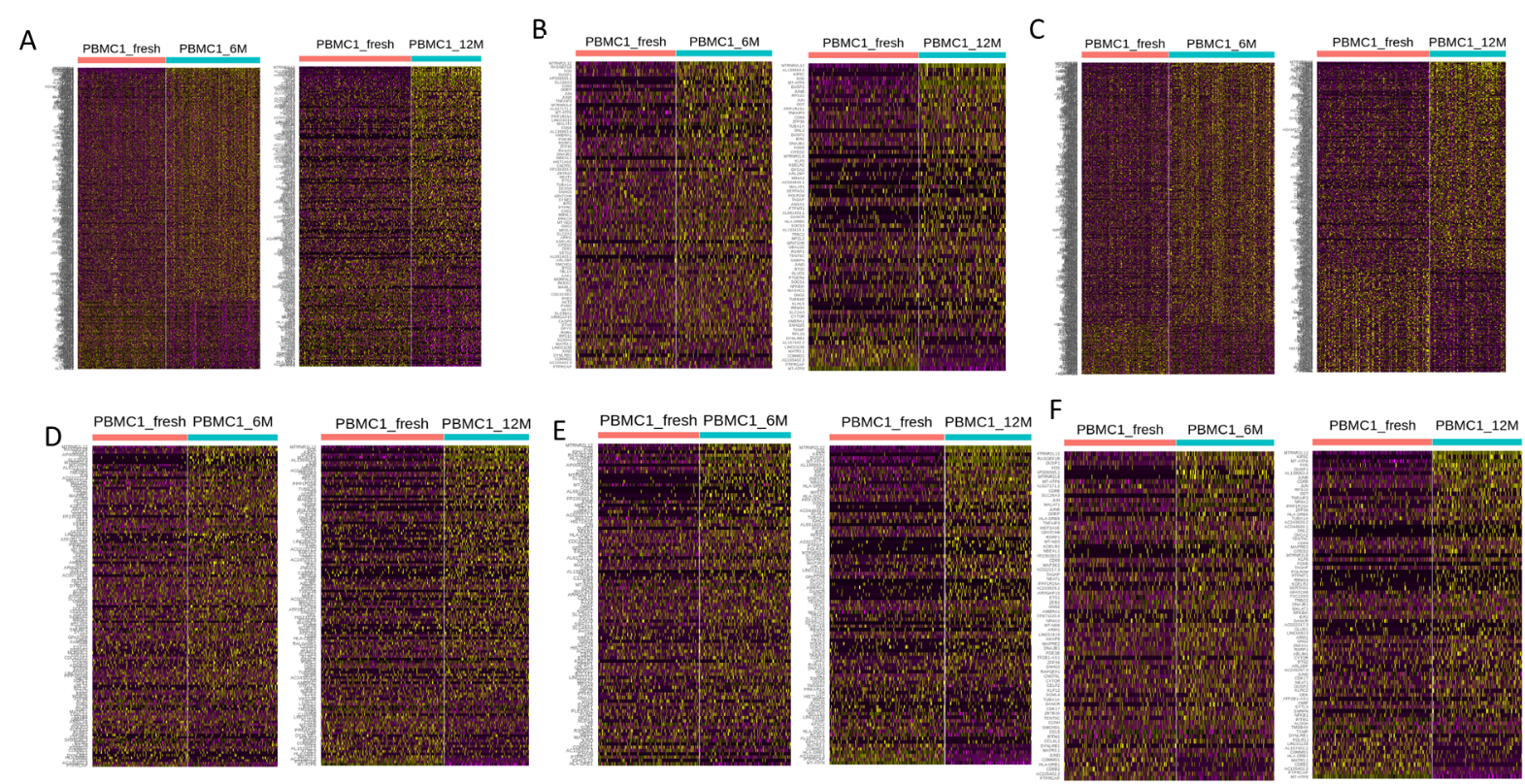
**

**Figure S4. Heatmap of DEGs identified from different cell types of donor 1 for different storage times versus fresh.** (A) Monocyte; (B) CD4+ T; (C) DCs; (D) NK cells; (E) B cells; (F) CD8+ T cells.


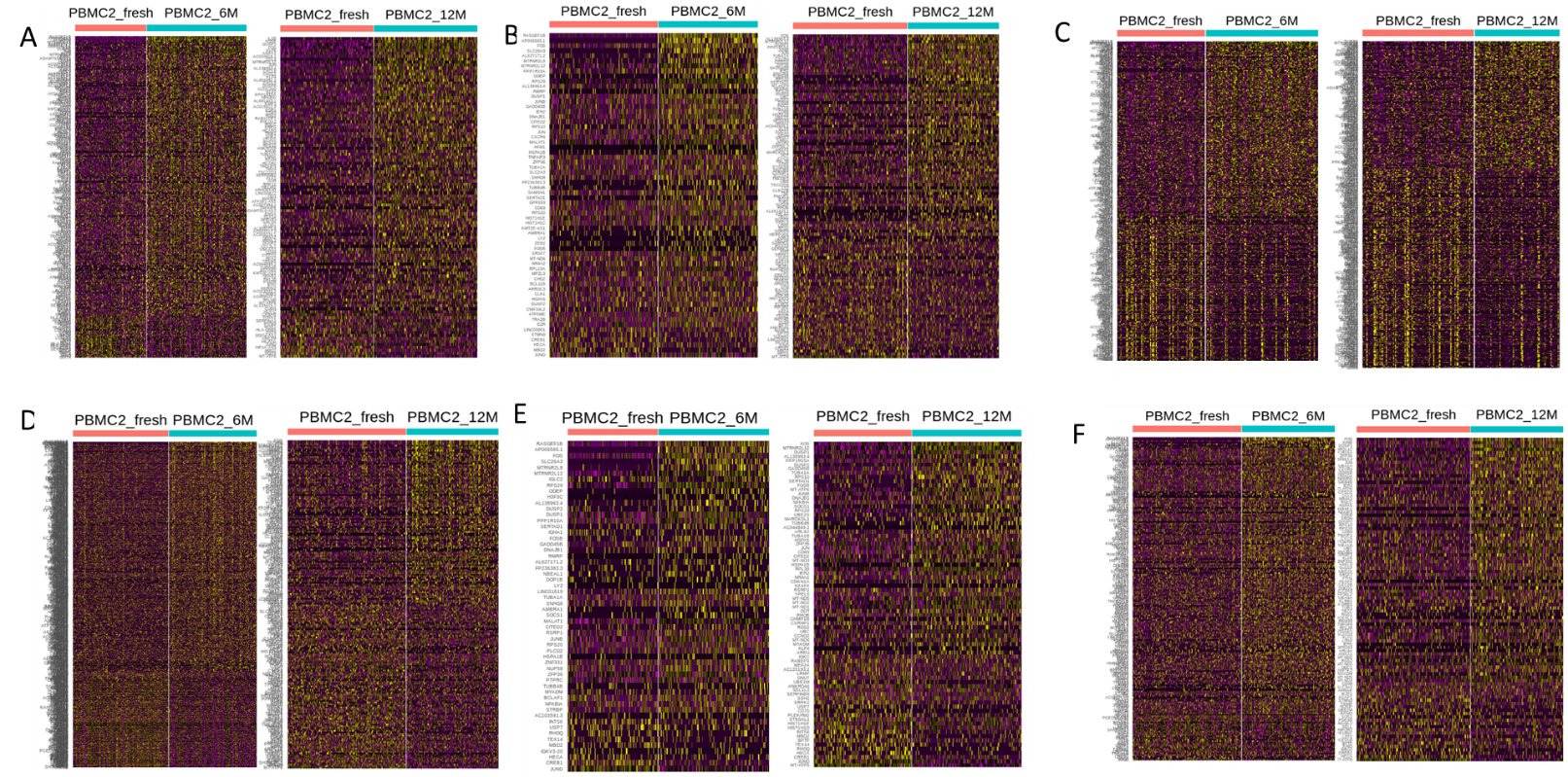


**Figure S5. Heatmap of DEGs identified from different cell types of donor 2 for different storage times versus fresh.** (A) Monocyte; (B) CD4+ T; (C) DCs; (D) NK cells; (E) B cells; (F) CD8+ T cells.

**
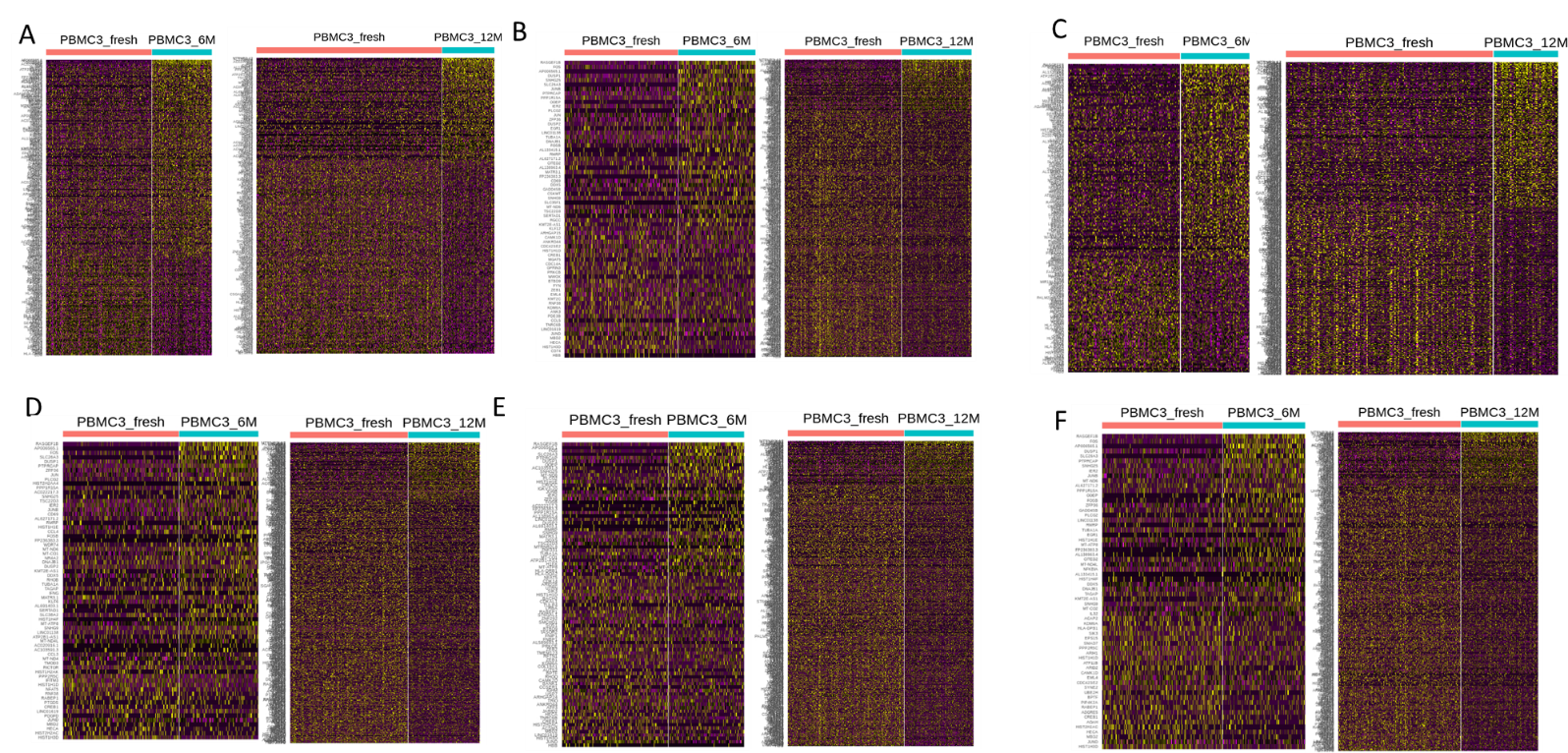
Figure S6. Heatmap of DEGs identified from different cell types of donor 3 for different storage times versus fresh.** (A) Monocyte; (B) CD4+ T; (C) DCs; (D) NK cells; (E) B cells; (F) CD8+ T cells.


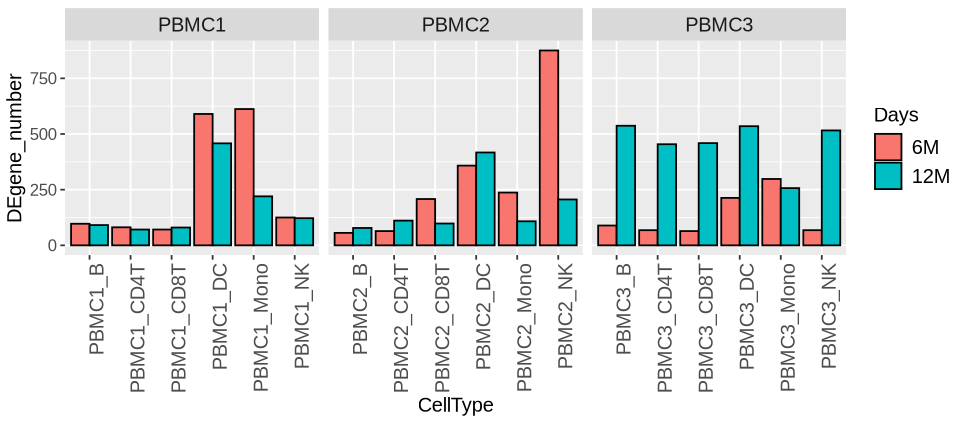


**Figure S7. Number of DEGs identified for each cell type of PBMCs cryopreserved for 6 months or 12 months vs. fresh PBMCs from each donor.** Bar plots depicting the number of DEGs.


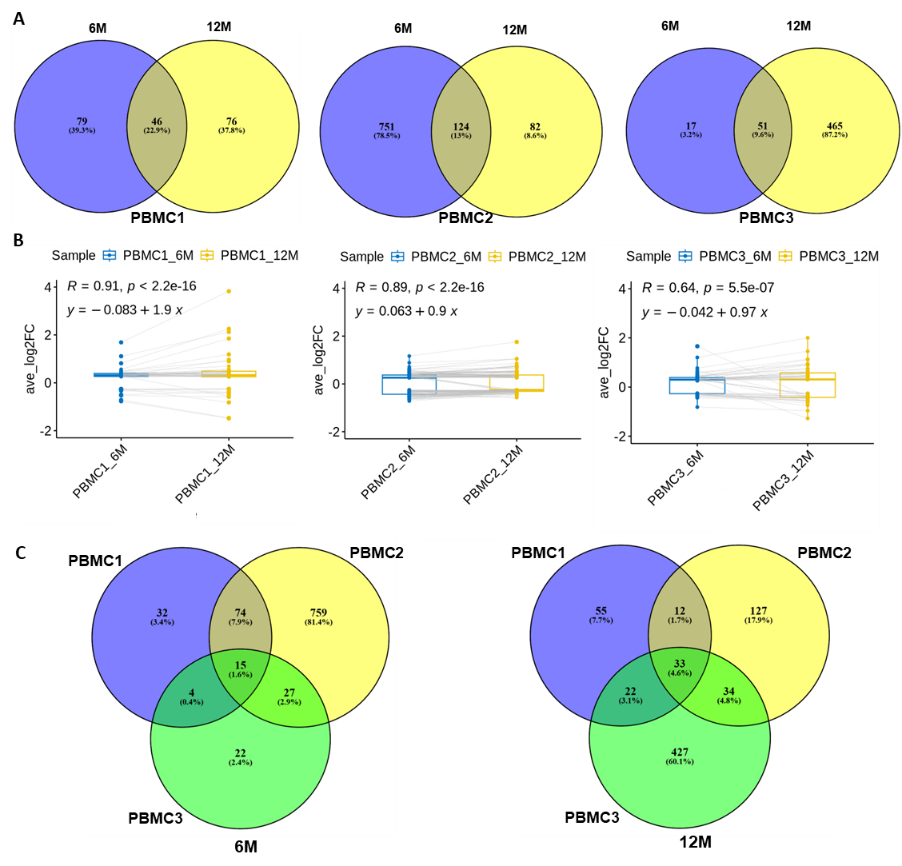

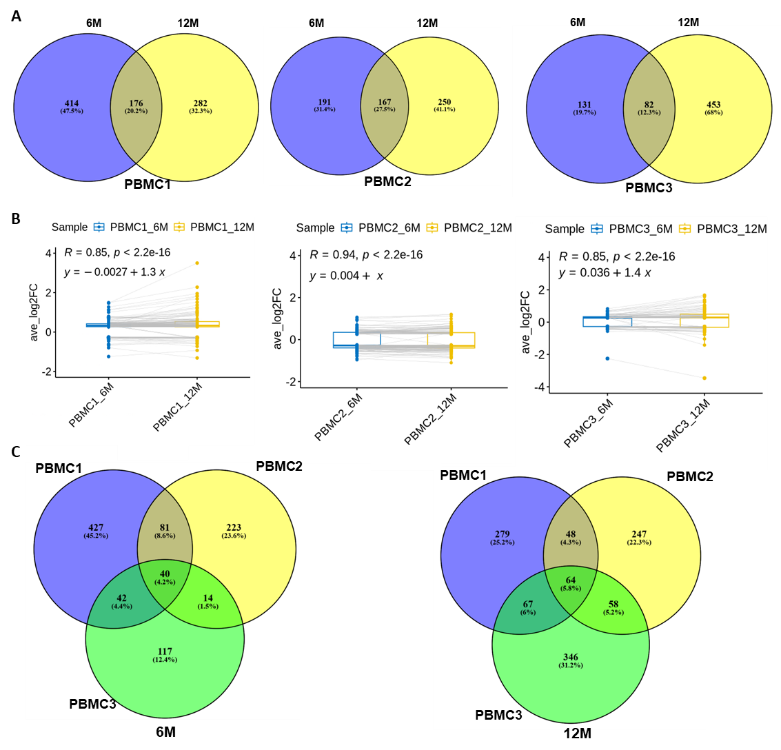
**Figure S8.** **DEG analysis of DCs with different cryopreservation times versus fresh.** (A). Venn Diagram analysis of DEGs identified from DCs of PBMCs cryopreserved for 6 and 12 months vs. fresh PMBCs for each donor. (B) Paired-boxplot of expression fold-change of common DEGs identified from DCs of PBMCs cryopreserved for 6 and 12 months vs. fresh PMBCs for each donor. The same gene was linked by a line. (C) Venn Diagram analysis of DEGs identified from DCs of PBMCs cryopreserved for different time points vs. fresh PMBCs for all three donors (Left: 6-month vs Fresh; Right: 12-month vs fresh).

**Figure S9.** **DEG analysis of NK with different cryopreservation times.** (A). Venn Diagram analysis of DEGs identified from NK cells of PBMCs cryopreserved for 6 and 12 months vs. fresh PMBCs for each donor. (B) Paired-boxplot of expression fold-change of common DEGs identified from NK cells of PBMCs cryopreserved for 6- and 12-months vs. fresh PMBCs for each donor. The same gene was linked by a line. (C) Venn Diagram of identified DEGs identified from NK cells of PBMCs cryopreserved for different time points vs. fresh PMBCs for all three donors (Left: 6-month vs Fresh; Right: 12-month vs fresh).

# Supplementary Tables

**Supplemental Table 1.** Quality of ScRNA-seq data

|  | **PBMC1_**  **fresh** | **PBMC2_**  **fresh** | **PBMC3_**  **fresh** | **PBMC1_6M** | **PBMC2_6M** | **PBMC3_6M** | **PBMC1_**  **12M** | **PBMC2_**  **12M** | **PBMC3_**  **12M** |
| --- | --- | --- | --- | --- | --- | --- | --- | --- | --- |
| Targeted Number of Cells | 10, 000 | 10, 000 | 10, 000 | 10, 000 | 10, 000 | 10, 000 | 10, 000 | 10, 000 | 10, 000 |
| Estimated Number of Cells before filtering | 12,029 | 11,138 | 13,759 | 11,010 | 11,564 | 8,803 | 8,522 | 9,550 | 6,982 |
| Estimated Number of Cells after filtering | 10,263 | 9,358 | 11,800 | 9,590 | 9,871 | 7,746 | 7,562 | 8,422 | 6,282 |
| Doublet rate | 9.24% | 9.03% | 9.30% | 9.05% | 10.65% | 10.03% | 7.26% | 7.36% | 7.99% |
| Mean Reads per Cell | 16,806 | 24,395 | 16,682 | 28,371 | 43,008 | 32,567 | 31,046 | 36,040 | 46,492 |
| Median Genes per Cell | 1,279 | 1,350 | 1,337 | 1,438 | 1,523 | 1,454 | 1,278 | 1,449 | 1,457 |
| Number of Reads | 202,154,629 | 271,715,929 | 229,534,144 | 312,366,497 | 497,338,750 | 286,691,374 | 264,574,826 | 344,179,265 | 324,608,029 |
| Valid Barcodes | 89.00% | 86.70% | 89.60% | 86.80% | 85.50% | 85.80% | 90.70% | 90.10% | 89.80% |
| Sequencing Saturation | 65.30% | 70.00% | 57.00% | 74.50% | 78.10% | 73.00% | 81.40% | 77.70% | 82.80% |
| Q30 Bases in Barcode | 93.30% | 93.30% | 93.00% | 93.90% | 93.90% | 94.10% | 94.00% | 94.10% | 94.20% |
| Q30 Bases in RNA Read | 92.30% | 92.50% | 90.60% | 87.40% | 86.60% | 87.30% | 92.50% | 92.20% | 93.10% |
| Q30 Bases in UMI | 93.20% | 93.30% | 93.00% | 92.40% | 92.50% | 92.80% | 93.80% | 93.80% | 93.80% |
| Reads Mapped to Genome | 95.60% | 95.50% | 90.30% | 93.10% | 91.10% | 89.90% | 92.30% | 91.70% | 94.40% |
| Reads Mapped Confidently to Transcriptome | 70.80% | 63.90% | 61.80% | 63.50% | 59.30% | 59.50% | 69.90% | 67.30% | 69.70% |
| Median percentage of mitochondrial genes | 2.86% | 3.33% | 2.50% | 3.11% | 3.37% | 2.99% | 2.65% | 3.63% | 3.10% |
| Fraction Reads in Cells | 97.40% | 96.60% | 96.10% | 96.80% | 95.30% | 97.00% | 95.30% | 95.80% | 96.60% |
| Total Genes Detected | 24,637 | 25,611 | 25,482 | 25,573 | 27,017 | 25,098 | 24,386 | 25,580 | 24,075 |
| Median UMI Counts per Cell | 3,285 | 3,771 | 3,594 | 3,675 | 4,286 | 4,174 | 3,235 | 4,301 | 4,441 |


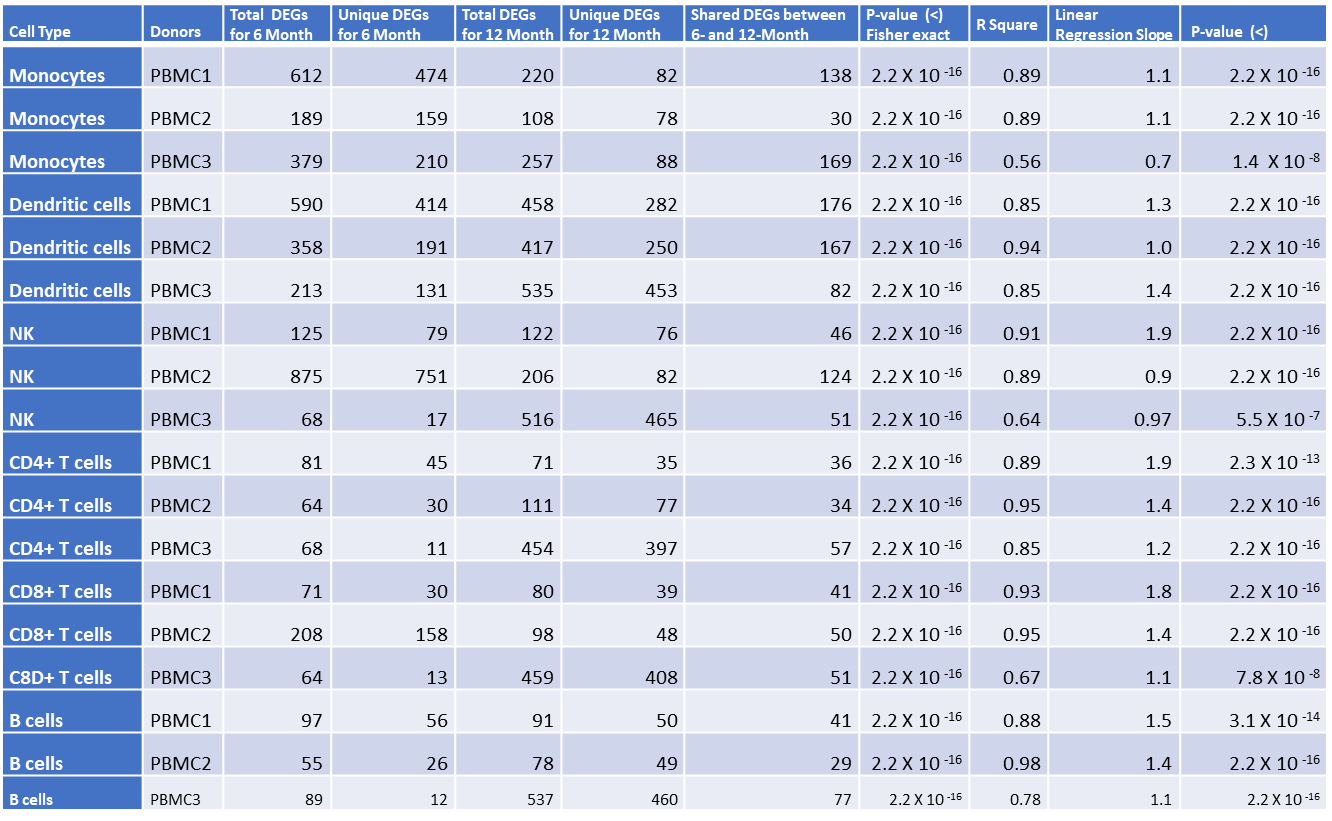
**Supplemental Table 2.** Summary of Differentially expressed genes (DEGs) in different cell types

**Supplemental Table 3.** DEGs identified for PBMCs cryopreserved for 6- and 12-months vs. fresh for each donor

| **Gene** | **6 months cryopreservation** | | | | | | **12 months cryopreservation** | | | | | | **Cell type** |
| --- | --- | --- | --- | --- | --- | --- | --- | --- | --- | --- | --- | --- | --- |
|  | **PBMC1 log2FC** | **PBMC1 padj** | **PBMC2 log2FC** | **PBMC2 padj** | **PBMC3 log2FC** | **PBMC3 padj** | **PBMC1 log2FC** | **PBMC1 padj** | **PBMC2 log2FC** | **PBMC2 padj** | **PBMC3 log2FC** | **PBMC3 padj** |  |
| AC007384.1 | 0.32 | 9.76E-09 | 0.36 | 1.08E-14 | 0.33 | 1.59E-21 | 0.38 | 1.00E-10 | 0.39 | 5.34E-24 | 0.29 | 4.14E-20 | Mono |
| AC009226.1 | 0.33 | 6.96E-08 | 0.30 | 3.93E-13 | 0.32 | 1.18E-17 | 0.56 | 5.41E-12 | 0.35 | 4.17E-26 | 0.26 | 6.22E-13 | Mono |
| AC015912.3 | 0.29 | 1 | 0.54 | 7.83E-23 | 0.26 | 6.90E-09 | 0.42 | 9.27E-04 | 0.66 | 3.90E-34 | 0.39 | 2.58E-20 | Mono |
| AC020916.1 | 1.37 | 3.34E-103 | 0.92 | 3.88E-84 | 1.10 | 1.31E-102 | 1.61 | 2.13E-97 | 1.13 | 8.21E-122 | 1.31 | 4.52E-93 | Mono |
| AL138963.4 | 0.31 | 4.00E-08 | 0.52 | 9.80E-24 | 0.34 | 3.34E-37 | 1.87 | 4.62E-58 | 1.06 | 8.98E-40 | 1.78 | 6.03E-179 | Mono |
| AL450992.3 | 0.53 | 2.71E-21 | 0.95 | 3.40E-86 | 0.50 | 2.97E-45 | 0.61 | 5.06E-21 | 0.87 | 2.10E-79 | 0.48 | 6.12E-44 | Mono |
| AL691403.1 | 0.44 | 4.73E-15 | 0.57 | 4.09E-35 | 0.45 | 1.34E-20 | 0.67 | 6.45E-19 | 0.70 | 4.73E-54 | 0.47 | 4.09E-21 | Mono |
| ANKRD28 | 0.32 | 1.46E-08 | 0.32 | 4.54E-24 | 0.33 | 2.52E-16 | 0.45 | 1.14E-12 | 0.46 | 4.37E-47 | 0.33 | 3.84E-26 | Mono |
| ANXA1 | 0.44 | 9.73E-08 | 0.71 | 2.22E-45 | 0.35 | 1.44E-07 | 0.58 | 3.48E-11 | 1.15 | 2.73E-114 | 0.47 | 2.28E-09 | Mono |
| ARHGEF40 | 0.40 | 7.59E-11 | 0.26 | 2.11E-05 | 0.29 | 7.58E-14 | 0.53 | 3.75E-11 | 0.41 | 5.15E-22 | 0.41 | 8.40E-20 | Mono |
| ATP2B1-AS1 | 0.28 | 1.09E-03 | 0.36 | 2.03E-25 | 0.81 | 1.26E-61 | 0.41 | 5.60E-12 | 0.39 | 4.61E-31 | 0.72 | 2.25E-36 | Mono |
| CCL3 | 0.93 | 1.55E-59 | 0.58 | 3.87E-29 | 0.52 | 5.83E-24 | 1.21 | 4.55E-82 | 1.10 | 3.29E-81 | 0.76 | 2.03E-49 | Mono |
| CD83 | 0.62 | 1.68E-24 | 0.64 | 1.17E-45 | 0.49 | 2.42E-26 | 0.55 | 1.02E-13 | 0.62 | 8.90E-43 | 0.30 | 3.50E-05 | Mono |
| CXCL2 | 0.55 | 7.78E-05 | 0.57 | 9.19E-12 | 0.62 | 4.47E-18 | 0.50 | 7.00E-03 | 1.03 | 3.11E-36 | 0.75 | 7.02E-35 | Mono |
| CXCL8 | 0.44 | 3.28E-12 | 0.63 | 1.01E-40 | 0.75 | 6.97E-26 | 0.59 | 1.54E-17 | 1.03 | 2.49E-83 | 1.23 | 4.33E-49 | Mono |
| DUSP1 | 1.07 | 8.30E-114 | 0.87 | 3.25E-145 | 0.84 | 7.60E-91 | 1.59 | 4.07E-145 | 1.33 | 1.43E-218 | 1.14 | 3.82E-87 | Mono |
| EREG | 0.84 | 2.64E-13 | 0.57 | 5.70E-17 | 0.42 | 1.36E-02 | 0.67 | 7.07E-10 | 0.51 | 3.57E-19 | 0.34 | 9.99E-07 | Mono |
| FCAR | 0.36 | 1.26E-02 | 0.46 | 1.88E-22 | 0.52 | 3.70E-21 | 0.30 | 1 | 0.45 | 7.40E-26 | 0.31 | 1.01E-04 | Mono |
| FOS | 1.23 | 3.25E-117 | 0.85 | 1.02E-140 | 1.02 | 1.06E-135 | 1.61 | 5.70E-140 | 1.20 | 4.68E-211 | 1.28 | 4.88E-114 | Mono |
| FOSB | 1.72 | 1.99E-163 | 1.33 | 8.75E-161 | 1.58 | 1.28E-193 | 1.65 | 6.29E-115 | 1.49 | 5.51E-186 | 1.57 | 4.95E-144 | Mono |
| IER2 | 0.39 | 1.48E-02 | 0.31 | 8.68E-08 | 0.35 | 1.07E-07 | 0.36 | 8.37E-01 | 0.28 | 8.74E-08 | 0.43 | 1.47E-07 | Mono |
| IL1B | 0.34 | 2.76E-19 | 0.91 | 2.14E-46 | 0.46 | 1.28E-17 | 0.56 | 2.94E-35 | 1.56 | 8.60E-118 | 0.62 | 9.76E-29 | Mono |
| JUN | 1.82 | 1.36E-107 | 0.83 | 3.49E-82 | 0.88 | 1.82E-48 | 2.20 | 2.64E-100 | 1.07 | 1.39E-108 | 1.29 | 5.56E-58 | Mono |
| KLF2 | 0.55 | 1.05E-31 | 0.38 | 2.13E-24 | 0.37 | 5.84E-10 | 1.10 | 1.90E-57 | 0.75 | 1.28E-73 | 0.74 | 1.44E-26 | Mono |
| KLF4 | 0.58 | 1.33E-22 | 0.60 | 6.70E-44 | 0.73 | 7.98E-57 | 0.72 | 1.69E-28 | 0.91 | 7.10E-96 | 0.79 | 1.38E-43 | Mono |
| KLF6 | 0.81 | 6.47E-65 | 0.80 | 4.26E-88 | 0.67 | 2.67E-49 | 0.98 | 2.10E-64 | 1.17 | 5.45E-157 | 0.68 | 9.90E-34 | Mono |
| LINC00877 | 0.53 | 3.49E-18 | 0.35 | 2.10E-20 | 0.35 | 4.58E-19 | 0.54 | 2.74E-18 | 0.41 | 1.08E-28 | 0.32 | 3.08E-13 | Mono |
| MTRNR2L12 | 1.68 | 2.09E-162 | 1.18 | 2.15E-165 | 0.41 | 6.74E-08 | 3.58 | 1.25E-245 | 1.09 | 1.50E-166 | 2.10 | 3.52E-167 | Mono |
| NR4A2 | 0.63 | 2.16E-26 | 0.36 | 1.18E-17 | 0.57 | 3.86E-34 | 0.64 | 7.18E-23 | 0.34 | 1.59E-18 | 0.60 | 3.84E-38 | Mono |
| PPP1R15A | 0.59 | 9.00E-35 | 0.43 | 4.09E-31 | 0.54 | 3.37E-30 | 0.84 | 1.71E-37 | 0.73 | 3.28E-80 | 0.77 | 4.32E-45 | Mono |
| RFX2 | 0.34 | 1.24E-03 | 0.33 | 1.04E-10 | 0.33 | 1.16E-06 | 0.34 | 4.11E-02 | 0.32 | 7.30E-09 | 0.34 | 3.28E-07 | Mono |
| RGCC | 0.29 | 2.39E-01 | 0.32 | 3.38E-11 | 0.62 | 1.53E-19 | 0.41 | 2.08E-02 | 0.53 | 6.57E-28 | 0.61 | 6.59E-22 | Mono |
| RPS10 | 0.43 | 2.42E-18 | 0.43 | 1.35E-32 | 0.33 | 7.94E-13 | 0.84 | 6.23E-50 | 0.48 | 2.81E-40 | 0.58 | 6.65E-33 | Mono |
| TNFAIP3 | 0.61 | 5.79E-15 | 0.38 | 1.36E-10 | 0.27 | 1.65E-04 | 0.57 | 8.72E-11 | 0.44 | 2.96E-22 | 0.36 | 9.94E-05 | Mono |
| TSC22D3 | 0.56 | 3.54E-15 | 0.32 | 8.53E-10 | 0.44 | 4.63E-15 | 0.94 | 3.46E-28 | 0.44 | 1.74E-20 | 0.64 | 1.10E-21 | Mono |
| VIM-AS1 | 0.53 | 4.24E-23 | 0.38 | 2.75E-22 | 0.33 | 5.04E-25 | 0.63 | 1.84E-20 | 0.32 | 9.80E-16 | 0.34 | 3.82E-27 | Mono |
| AL138963.4 | 0.41 | 1.98E-55 | 0.58 | 1.07E-46 | 0.34 | 6.75E-58 | 2.08 | 6.38E-262 | 1.05 | 7.39E-80 | 2.06 | 0 | CD4T |
| CD69 | 0.54 | 1.81E-55 | 0.33 | 1.39E-25 | 0.32 | 3.75E-25 | 0.65 | 4.66E-71 | 0.42 | 8.39E-43 | 0.47 | 1.04E-57 | CD4T |
| CITED2 | 0.28 | 7.12E-13 | 0.45 | 5.56E-35 | 0.35 | 1.28E-23 | 0.45 | 2.07E-20 | 0.70 | 4.83E-79 | 0.74 | 4.43E-90 | CD4T |
| DNAJB1 | 0.37 | 7.76E-11 | 0.45 | 5.02E-43 | 0.40 | 8.01E-36 | 0.54 | 7.01E-21 | 0.62 | 5.28E-74 | 0.77 | 1.44E-114 | CD4T |
| DUSP1 | 0.83 | 4.78E-135 | 0.53 | 1.96E-72 | 0.80 | 7.98E-107 | 1.51 | 0 | 1.02 | 6.01E-172 | 1.43 | 1.23E-288 | CD4T |
| FOS | 1.05 | 5.71E-158 | 1.06 | 2.50E-122 | 1.17 | 3.46E-114 | 1.85 | 0 | 1.80 | 2.65E-259 | 2.06 | 0 | CD4T |
| FOSB | 0.43 | 6.94E-42 | 0.30 | 6.37E-35 | 0.39 | 1.09E-31 | 0.51 | 7.03E-54 | 0.45 | 1.69E-67 | 0.51 | 2.59E-63 | CD4T |
| IER2 | 0.31 | 5.06E-05 | 0.47 | 5.56E-38 | 0.55 | 3.42E-30 | 0.55 | 4.96E-12 | 0.62 | 2.95E-63 | 0.96 | 8.46E-75 | CD4T |
| JUN | 0.54 | 7.12E-39 | 0.41 | 1.06E-45 | 0.52 | 7.28E-41 | 0.89 | 8.34E-77 | 0.85 | 1.24E-117 | 0.84 | 5.01E-82 | CD4T |
| JUNB | 0.53 | 9.45E-56 | 0.52 | 5.81E-46 | 0.66 | 1.15E-91 | 0.96 | 1.84E-142 | 0.86 | 2.62E-98 | 1.33 | 3.28E-279 | CD4T |
| JUND | -0.35 | 8.71E-17 | -0.54 | 1.16E-11 | -0.42 | 5.77E-42 | 0.29 | 1.10E-30 | -0.42 | 4.70E-01 | -0.56 | 1.35E-63 | CD4T |
| PPP1R15A | 0.45 | 1.71E-40 | 0.62 | 1.26E-50 | 0.63 | 6.10E-79 | 0.78 | 2.70E-90 | 1.02 | 1.73E-129 | 1.03 | 8.20E-182 | CD4T |
| TUBA1A | 0.32 | 1.09E-13 | 0.38 | 1.57E-27 | 0.41 | 1.69E-32 | 0.64 | 4.82E-51 | 0.73 | 1.76E-93 | 0.78 | 5.07E-91 | CD4T |
| ZFP36 | 0.38 | 3.55E-28 | 0.38 | 9.88E-27 | 0.49 | 1.60E-53 | 0.64 | 1.73E-49 | 0.65 | 3.20E-66 | 0.85 | 1.30E-117 | CD4T |
| DNAJB1 | 0.28 | 3.35E-02 | 0.39 | 9.62E-16 | 0.27 | 2.51E-03 | 0.35 | 1.02E-05 | 0.39 | 7.03E-27 | 0.60 | 7.12E-29 | CD8T |
| DUSP1 | 0.93 | 1.47E-82 | 0.64 | 3.46E-48 | 0.83 | 1.59E-32 | 1.68 | 9.61E-233 | 1.10 | 5.88E-94 | 1.56 | 7.69E-123 | CD8T |
| FOS | 0.91 | 1.45E-48 | 1.24 | 2.88E-77 | 1.25 | 4.31E-39 | 1.74 | 1.12E-153 | 1.94 | 2.36E-142 | 2.21 | 1.23E-127 | CD8T |
| JUNB | 0.46 | 1.44E-12 | 0.64 | 1.49E-29 | 0.57 | 8.18E-19 | 1.06 | 3.15E-74 | 1.13 | 3.86E-67 | 1.34 | 9.14E-95 | CD8T |
| JUND | -0.36 | 2.48E-13 | -0.42 | 1.00E+00 | -0.49 | 5.96E-18 | 0.30 | 3.68E-14 | -0.39 | 1 | -0.63 | 3.82E-27 | CD8T |
| PPP1R15A | 0.31 | 3.64E-09 | 0.66 | 3.28E-34 | 0.50 | 9.30E-14 | 0.61 | 6.85E-36 | 1.06 | 2.26E-76 | 0.97 | 1.76E-52 | CD8T |
| TAGAP | 0.33 | 6.57E-09 | 0.29 | 9.42E-09 | 0.26 | 2.25E-02 | 0.44 | 7.39E-13 | 0.38 | 1.08E-10 | 0.45 | 4.24E-14 | CD8T |
| TUBA1A | 0.26 | 1.80E-04 | 0.46 | 5.09E-21 | 0.38 | 1.85E-05 | 0.57 | 7.17E-26 | 0.72 | 4.90E-52 | 0.84 | 9.30E-33 | CD8T |
| ZFP36 | 0.27 | 1.09E-04 | 0.55 | 2.85E-27 | 0.44 | 3.95E-15 | 0.60 | 1.02E-28 | 0.91 | 9.89E-56 | 0.93 | 7.10E-54 | CD8T |
| AL138963.4 | 0.30 | 4.12E-08 | 0.49 | 7.64E-18 | 0.34 | 2.00E-24 | 1.60 | 7.86E-51 | 0.89 | 1.07E-31 | 1.49 | 9.99E-177 | B |
| DUSP1 | 0.91 | 3.31E-47 | 0.47 | 7.68E-27 | 0.69 | 1.40E-51 | 1.81 | 6.61E-118 | 0.90 | 1.86E-88 | 1.49 | 3.50E-213 | B |
| DUSP2 | 0.38 | 9.81E-01 | 0.48 | 4.80E-10 | 0.34 | 6.31E-06 | 0.35 | 1 | 0.74 | 1.59E-29 | 0.53 | 7.01E-26 | B |
| FOS | 1.56 | 1.39E-67 | 1.06 | 2.46E-64 | 1.10 | 1.37E-80 | 2.96 | 5.01E-159 | 1.67 | 3.35E-138 | 2.37 | 6.59E-287 | B |
| FOSB | 0.47 | 9.47E-21 | 0.43 | 6.20E-18 | 0.38 | 1.67E-28 | 0.66 | 2.69E-29 | 0.61 | 2.33E-31 | 0.62 | 4.38E-71 | B |
| JUNB | 0.41 | 7.20E-08 | 0.30 | 3.59E-07 | 0.40 | 4.52E-17 | 0.97 | 5.51E-35 | 0.59 | 1.28E-26 | 0.88 | 6.47E-75 | B |
| MBD2 | -0.26 | 1 | -0.36 | 1.35E-11 | -0.42 | 2.13E-14 | -0.28 | 1 | -0.40 | 3.70E-13 | -0.57 | 1.43E-25 | B |
| ZFP36 | 0.33 | 1.03E-03 | 0.27 | 2.35E-06 | 0.40 | 8.26E-22 | 0.51 | 1.37E-08 | 0.37 | 8.36E-14 | 0.66 | 5.00E-52 | B |
| CD69 | 0.28 | 1.67E-03 | 0.32 | 2.10E-02 | 0.44 | 5.44E-09 | 0.57 | 2.60E-11 | 0.39 | 2.89E-04 | 0.65 | 2.40E-27 | NK |
| DUSP1 | 1.11 | 1.10E-52 | 0.58 | 3.99E-12 | 0.77 | 7.49E-29 | 2.12 | 7.77E-131 | 1.06 | 7.36E-26 | 1.46 | 2.70E-112 | NK |
| FOS | 0.82 | 1.11E-25 | 1.16 | 6.95E-17 | 1.20 | 1.13E-40 | 2.26 | 6.55E-106 | 1.76 | 3.50E-36 | 2.00 | 3.80E-127 | NK |
| JUN | 0.46 | 1.43E-12 | 0.71 | 2.24E-09 | 0.58 | 6.78E-08 | 1.19 | 6.90E-40 | 1.05 | 4.50E-14 | 0.95 | 3.54E-20 | NK |
| JUNB | 0.29 | 1 | 0.32 | 1.00E+00 | 0.45 | 3.18E-08 | 0.96 | 2.61E-24 | 0.67 | 6.84E-07 | 0.93 | 1.31E-40 | NK |
| KLF6 | 0.29 | 1 | 0.49 | 1.75E-04 | 0.30 | 1.31E-01 | 0.50 | 1.19E-06 | 0.53 | 2.28E-05 | 0.48 | 3.80E-10 | NK |
| NR4A2 | 0.51 | 3.37E-13 | 0.47 | 5.22E-01 | 0.33 | 6.96E-06 | 0.90 | 2.07E-36 | 0.54 | 1.11E-03 | 0.53 | 2.89E-21 | NK |
| AC007384.1 | 0.32 | 1 | 0.36 | 1.00E+00 | 0.40 | 1.27E-01 | 0.54 | 6.76E-05 | 0.40 | 1 | 0.50 | 4.49E-04 | DC |
| AC020916.1 | 0.61 | 4.58E-04 | 0.35 | 1.00E+00 | 0.40 | 1.07E-01 | 1.24 | 6.29E-16 | 0.52 | 1 | 0.84 | 8.02E-08 | DC |
| AC022217.3 | 0.31 | 1 | 0.44 | 2.40E-03 | 0.50 | 1.26E-08 | 0.46 | 4.14E-02 | 0.41 | 4.06E-04 | 0.28 | 2.73E-03 | DC |
| AL133415.1 | 0.57 | 6.87E-01 | 0.37 | 1.00E+00 | 0.69 | 1.41E-06 | 1.19 | 1.10E-05 | 0.30 | 1 | 1.30 | 1.14E-09 | DC |
| AL138963.4 | 0.27 | 1 | 0.57 | 1.00E+00 | 0.29 | 1 | 1.83 | 3.15E-14 | 0.60 | 1 | 1.67 | 8.03E-21 | DC |
| AL691403.1 | 0.56 | 5.17E-07 | 0.55 | 7.42E-05 | 0.48 | 1.47E-05 | 0.90 | 7.45E-13 | 0.61 | 3.47E-05 | 0.94 | 4.67E-14 | DC |
| ATP2B1-AS1 | 0.46 | 1 | 0.26 | 1.00E+00 | 0.64 | 2.19E-02 | 0.84 | 4.66E-03 | 0.54 | 1 | 1.06 | 1.80E-05 | DC |
| CXCL8 | 0.67 | 1 | 0.35 | 1.00E+00 | 0.64 | 4.38E-03 | 1.00 | 5.43E-02 | 0.59 | 1 | 1.25 | 1.94E-05 | DC |
| DDIT4 | -0.26 | 1 | -0.41 | 1.00E+00 | -0.57 | 1 | -0.26 | 1 | -0.35 | 1 | -0.54 | 1 | DC |
| DUSP1 | 1.49 | 8.17E-10 | 1.03 | 8.90E-09 | 0.83 | 1.91E-03 | 2.28 | 1.87E-17 | 1.21 | 8.86E-08 | 1.65 | 9.88E-11 | DC |
| ELF1 | 0.58 | 6.57E-06 | 0.25 | 1.00E+00 | 0.38 | 8.67E-01 | 0.58 | 6.67E-03 | 0.28 | 1 | 0.38 | 1 | DC |
| EREG | 0.76 | 1 | 0.36 | 1.00E+00 | 0.25 | 1 | 0.96 | 1 | 0.44 | 1 | 0.36 | 1 | DC |
| FOS | 1.13 | 2.07E-05 | 0.96 | 3.98E-07 | 0.68 | 7.73E-02 | 1.82 | 4.91E-18 | 1.19 | 2.08E-09 | 1.58 | 9.21E-10 | DC |
| FOSB | 1.01 | 1.27E-15 | 0.50 | 9.92E-01 | 0.71 | 3.63E-07 | 1.51 | 7.87E-27 | 0.76 | 4.80E-03 | 1.37 | 1.68E-15 | DC |
| KLF4 | 0.31 | 1 | 0.32 | 1.00E+00 | 0.30 | 1 | 0.69 | 1.33E-02 | 0.55 | 1 | 0.78 | 4.63E-05 | DC |
| KLF6 | 0.41 | 1 | 0.39 | 1.00E+00 | 0.44 | 1 | 0.88 | 2.93E-08 | 0.78 | 2.44E-05 | 1.02 | 3.23E-05 | DC |
| NR4A2 | 0.67 | 3.22E-01 | 0.47 | 5.78E-01 | 0.27 | 1 | 0.91 | 1.95E-05 | 0.58 | 6.48E-01 | 0.80 | 5.94E-04 | DC |

**Supplemental Table 4.** Enrichment analysis of common DEGs in Monocyte

| **Category** | **Term** | **Count** | **List Total** | **Fold Enrichment** | **Benjamini** | **FDR** |
| --- | --- | --- | --- | --- | --- | --- |
| GOTERM_BP_FAT | GO:0034097~response to cytokine | 12 | 25 | 10.11 | 2.61E-06 | 0.000235 |
| GOTERM_BP_FAT | GO:1901652~response to peptide | 12 | 25 | 9.95 | 2.61E-06 | 0.000235 |
| GOTERM_BP_FAT | GO:0010557~positive regulation of macromolecule biosynthetic process | 17 | 25 | 4.84 | 2.61E-06 | 0.000235 |
| KEGG_PATHWAY | hsa04657:IL-17 signaling pathway | 7 | 17 | 38.42 | 9.77E-07 | 7.11E-05 |
| GOTERM_BP_FAT | GO:0009891~positive regulation of biosynthetic process | 17 | 25 | 4.64 | 3.72E-06 | 0.000334 |
| GOTERM_BP_FAT | GO:1901700~response to oxygen-containing compound | 14 | 25 | 6.44 | 4.67E-06 | 0.000419 |
| GOTERM_BP_FAT | GO:0071345~cellular response to cytokine stimulus | 11 | 25 | 10.43 | 4.84E-06 | 0.000435 |
| GOTERM_BP_FAT | GO:0010604~positive regulation of macromolecule metabolic process | 18 | 25 | 3.91 | 6.17E-06 | 0.000554 |
| GOTERM_BP_FAT | GO:0033993~response to lipid | 11 | 25 | 9.40 | 9.67E-06 | 0.000868 |
| GOTERM_BP_FAT | GO:0009893~positive regulation of metabolic process | 18 | 25 | 3.59 | 1.82E-05 | 0.001632 |
| GOTERM_BP_FAT | GO:0070887~cellular response to chemical stimulus | 14 | 25 | 5.16 | 3.31E-05 | 0.002975 |
| GOTERM_BP_FAT | GO:0034599~cellular response to oxidative stress | 7 | 25 | 22.38 | 5.26E-05 | 0.004720 |
| GOTERM_BP_FAT | GO:0006950~response to stress | 17 | 25 | 3.59 | 5.26E-05 | 0.004720 |
| GOTERM_BP_FAT | GO:1901701~cellular response to oxygen-containing compound | 11 | 25 | 7.37 | 5.76E-05 | 0.005173 |
| KEGG_PATHWAY | hsa05323:Rheumatoid arthritis | 6 | 17 | 32.93 | 2.60E-05 | 0.001897 |
| GOTERM_BP_FAT | GO:0034614~cellular response to reactive oxygen species | 6 | 25 | 32.46 | 8.25E-05 | 0.007404 |
| GOTERM_BP_FAT | GO:0010468~regulation of gene expression | 20 | 25 | 2.68 | 8.25E-05 | 0.007404 |
| GOTERM_BP_FAT | GO:0010556~regulation of macromolecule biosynthetic process | 20 | 25 | 2.63 | 0.000110 | 0.009857 |
| GOTERM_BP_FAT | GO:0045944~positive regulation of transcription by RNA polymerase II | 11 | 25 | 6.57 | 0.000127 | 0.011411 |
| GOTERM_BP_FAT | GO:0062197~cellular response to chemical stress | 7 | 25 | 17.75 | 0.000129 | 0.011599 |
| GOTERM_BP_FAT | GO:0002682~regulation of immune system process | 12 | 25 | 5.31 | 0.000207 | 0.018554 |
| WIKIPATHWAYS | WP5353:Macrophage stimulating protein MSP signaling | 6 | 20 | 24.30 | 0.000500 | 0.044987 |
| GOTERM_BP_FAT | GO:2001141~regulation of RNA biosynthetic process | 16 | 25 | 3.40 | 0.000211 | 0.018916 |
| GOTERM_BP_FAT | GO:0002684~positive regulation of immune system process | 10 | 25 | 7.21 | 0.000211 | 0.018916 |
| GOTERM_BP_FAT | GO:0000302~response to reactive oxygen species | 6 | 25 | 24.17 | 0.000227 | 0.020409 |
| GOTERM_BP_FAT | GO:0071396~cellular response to lipid | 8 | 25 | 10.99 | 0.000250 | 0.022456 |
| GOTERM_BP_FAT | GO:0071621~granulocyte chemotaxis | 5 | 25 | 43.46 | 0.000259 | 0.023259 |
| GOTERM_BP_FAT | GO:0006979~response to oxidative stress | 7 | 25 | 13.96 | 0.000373 | 0.033458 |
| GOTERM_BP_FAT | GO:0097530~granulocyte migration | 5 | 25 | 37.55 | 0.000428 | 0.038430 |
| GOTERM_BP_FAT | GO:0014070~response to organic cyclic compound | 9 | 25 | 7.75 | 0.000440 | 0.039489 |
| GOTERM_BP_FAT | GO:0070555~response to interleukin-1 | 5 | 25 | 36.15 | 0.000451 | 0.040530 |
| GOTERM_BP_FAT | GO:0001817~regulation of cytokine production | 9 | 25 | 7.65 | 0.000451 | 0.040530 |
| GOTERM_BP_FAT | GO:0042981~regulation of apoptotic process | 11 | 25 | 5.36 | 0.000456 | 0.040983 |
| GOTERM_BP_FAT | GO:0051252~regulation of RNA metabolic process | 16 | 25 | 3.10 | 0.000486 | 0.043666 |
| WIKIPATHWAYS | WP5115:Network map of SARS CoV 2 signaling | 7 | 20 | 12.06 | 0.001053 | 0.094724 |
| GOTERM_BP_FAT | GO:0036230~granulocyte activation | 4 | 25 | 85.95 | 0.000546 | 0.049059 |
| GOTERM_BP_FAT | GO:0043067~regulation of programmed cell death | 11 | 25 | 5.19 | 0.000552 | 0.049605 |
| GOTERM_BP_FAT | GO:0006355~regulation of DNA-templated transcription | 15 | 25 | 3.21 | 0.000812 | 0.072907 |
| GOTERM_BP_FAT | GO:0045893~positive regulation of DNA-templated transcription | 11 | 25 | 4.90 | 0.000841 | 0.075512 |
| GOTERM_BP_FAT | GO:1902680~positive regulation of RNA biosynthetic process | 11 | 25 | 4.90 | 0.000841 | 0.075512 |
| GOTERM_BP_FAT | GO:0045935~positive regulation of nucleobase-containing compound metabolic process | 12 | 25 | 4.27 | 0.000891 | 0.079969 |
| GOTERM_BP_FAT | GO:0019219~regulation of nucleobase-containing compound metabolic process | 16 | 25 | 2.87 | 0.001034 | 0.092799 |
| GOTERM_BP_FAT | GO:0097529~myeloid leukocyte migration | 5 | 25 | 27.05 | 0.001043 | 0.093626 |
| KEGG_PATHWAY | hsa05142:Chagas disease | 5 | 17 | 25.31 | 0.000724 | 0.052690 |
| GOTERM_MF_FAT | GO:0001228~DNA-binding transcription activator activity, RNA polymerase II-specific | 7 | 23 | 10.51 | 0.002848 | 0.247416 |
| KEGG_PATHWAY | hsa05417:Lipid and atherosclerosis | 6 | 17 | 14.49 | 0.000724 | 0.052690 |
| GOTERM_MF_FAT | GO:0001216~DNA-binding transcription activator activity | 7 | 23 | 10.36 | 0.002848 | 0.247416 |
| GOTERM_BP_FAT | GO:0010605~negative regulation of macromolecule metabolic process | 14 | 25 | 3.34 | 0.001171 | 0.105136 |
| GOTERM_BP_FAT | GO:0001775~cell activation | 8 | 25 | 7.93 | 0.001171 | 0.105136 |
| KEGG_PATHWAY | hsa04620:Toll-like receptor signaling pathway | 5 | 17 | 23.92 | 0.000724 | 0.052690 |
| GOTERM_BP_FAT | GO:1901698~response to nitrogen compound | 9 | 25 | 6.31 | 0.001261 | 0.113242 |
| GOTERM_BP_FAT | GO:0006357~regulation of transcription by RNA polymerase II | 13 | 25 | 3.63 | 0.001261 | 0.113242 |
| GOTERM_BP_FAT | GO:0030595~leukocyte chemotaxis | 5 | 25 | 24.95 | 0.001267 | 0.113790 |
| GOTERM_BP_FAT | GO:0071356~cellular response to tumor necrosis factor | 5 | 25 | 24.17 | 0.001403 | 0.125959 |
| WIKIPATHWAYS | WP75:Toll like receptor signaling | 5 | 20 | 23.02 | 0.002812 | 0.252894 |
| KEGG_PATHWAY | hsa04668:TNF signaling pathway | 5 | 17 | 21.91 | 0.000851 | 0.061993 |
| GOTERM_BP_FAT | GO:0030593~neutrophil chemotaxis | 4 | 25 | 51.57 | 0.001752 | 0.157271 |
| GOTERM_BP_FAT | GO:2000026~regulation of multicellular organismal development | 10 | 25 | 5.02 | 0.001752 | 0.157271 |
| GOTERM_BP_FAT | GO:0051254~positive regulation of RNA metabolic process | 11 | 25 | 4.32 | 0.001869 | 0.167839 |
| GOTERM_BP_FAT | GO:0032496~response to lipopolysaccharide | 6 | 25 | 13.22 | 0.001872 | 0.168105 |
| GOTERM_BP_FAT | GO:0006952~defense response | 10 | 25 | 4.92 | 0.001935 | 0.173775 |
| REACTOME_PATHWAY | R-HSA-6783783~Interleukin-10 signaling | 4 | 20 | 47.46 | 0.009635 | 0.942814 |
| GOTERM_BP_FAT | GO:0009892~negative regulation of metabolic process | 14 | 25 | 3.12 | 0.002004 | 0.179910 |
| GOTERM_BP_FAT | GO:0006954~inflammatory response | 7 | 25 | 8.98 | 0.002140 | 0.192170 |
| GOTERM_BP_FAT | GO:0034612~response to tumor necrosis factor | 5 | 25 | 20.79 | 0.002140 | 0.192170 |
| GOTERM_BP_FAT | GO:0045765~regulation of angiogenesis | 6 | 25 | 12.58 | 0.002152 | 0.193258 |
| GOTERM_BP_FAT | GO:0032663~regulation of interleukin-2 production | 4 | 25 | 45.50 | 0.002174 | 0.195195 |
| GOTERM_BP_FAT | GO:0002237~response to molecule of bacterial origin | 6 | 25 | 12.44 | 0.002184 | 0.196087 |
| REACTOME_PATHWAY | R-HSA-449147~Signaling by Interleukins | 7 | 20 | 8.47 | 0.009635 | 0.942814 |
| GOTERM_BP_FAT | GO:1901342~regulation of vasculature development | 6 | 25 | 12.28 | 0.002285 | 0.205135 |
| GOTERM_BP_FAT | GO:0070301~cellular response to hydrogen peroxide | 4 | 25 | 43.58 | 0.002306 | 0.207013 |
| GOTERM_BP_FAT | GO:1990266~neutrophil migration | 4 | 25 | 43.58 | 0.002306 | 0.207013 |
| KEGG_PATHWAY | hsa05418:Fluid shear stress and atherosclerosis | 5 | 17 | 18.49 | 0.001416 | 0.103074 |
| GOTERM_BP_FAT | GO:0022603~regulation of anatomical structure morphogenesis | 8 | 25 | 6.67 | 0.002412 | 0.216548 |
| GOTERM_BP_FAT | GO:0001818~negative regulation of cytokine production | 6 | 25 | 11.78 | 0.002555 | 0.229380 |
| GOTERM_BP_FAT | GO:0010628~positive regulation of gene expression | 9 | 25 | 5.41 | 0.002555 | 0.229380 |
| GOTERM_BP_FAT | GO:0009719~response to endogenous stimulus | 9 | 25 | 5.37 | 0.002640 | 0.237065 |
| WIKIPATHWAYS | WP2431:Spinal cord injury | 5 | 20 | 18.23 | 0.005257 | 0.472834 |
| GOTERM_BP_FAT | GO:0071347~cellular response to interleukin-1 | 4 | 25 | 39.67 | 0.002813 | 0.252574 |
| GOTERM_BP_FAT | GO:0033554~cellular response to stress | 10 | 25 | 4.53 | 0.002830 | 0.254062 |
| GOTERM_BP_FAT | GO:0071222~cellular response to lipopolysaccharide | 5 | 25 | 17.74 | 0.003143 | 0.282154 |
| GOTERM_BP_FAT | GO:0007166~cell surface receptor signaling pathway | 11 | 25 | 3.91 | 0.003143 | 0.282154 |
| GOTERM_BP_FAT | GO:0060326~cell chemotaxis | 5 | 25 | 17.19 | 0.003478 | 0.312280 |
| GOTERM_BP_FAT | GO:0071219~cellular response to molecule of bacterial origin | 5 | 25 | 16.67 | 0.003855 | 0.346145 |
| GOTERM_BP_FAT | GO:0010558~negative regulation of macromolecule biosynthetic process | 12 | 25 | 3.39 | 0.004064 | 0.364901 |
| GOTERM_BP_FAT | GO:1990869~cellular response to chemokine | 4 | 25 | 32.57 | 0.004487 | 0.402867 |
| GOTERM_BP_FAT | GO:1990868~response to chemokine | 4 | 25 | 32.57 | 0.004487 | 0.402867 |
| GOTERM_BP_FAT | GO:0009890~negative regulation of biosynthetic process | 12 | 25 | 3.30 | 0.004948 | 0.444224 |
| GOTERM_BP_FAT | GO:0050900~leukocyte migration | 5 | 25 | 15.17 | 0.005160 | 0.463259 |
| GOTERM_BP_FAT | GO:0048468~cell development | 11 | 25 | 3.62 | 0.005406 | 0.485364 |
| GOTERM_BP_FAT | GO:0071216~cellular response to biotic stimulus | 5 | 25 | 14.82 | 0.005489 | 0.492855 |
| GOTERM_BP_FAT | GO:0042542~response to hydrogen peroxide | 4 | 25 | 29.47 | 0.005636 | 0.506051 |
| KEGG_PATHWAY | hsa04621:NOD-like receptor signaling pathway | 5 | 17 | 13.80 | 0.003836 | 0.279331 |
| GOTERM_MF_FAT | GO:0098772~molecular function regulator activity | 11 | 23 | 3.48 | 0.019194 | 1.667717 |
| WIKIPATHWAYS | WP3624:Lung fibrosis | 4 | 20 | 27.77 | 0.012005 | 1.079848 |
| KEGG_PATHWAY | hsa05133:Pertussis | 4 | 17 | 26.74 | 0.003867 | 0.281605 |
| GOTERM_BP_FAT | GO:0008285~negative regulation of cell population proliferation | 7 | 25 | 6.69 | 0.007117 | 0.638966 |
| GOTERM_BP_FAT | GO:0051726~regulation of cell cycle | 8 | 25 | 5.35 | 0.007142 | 0.641277 |
| GOTERM_BP_FAT | GO:0097305~response to alcohol | 5 | 25 | 13.43 | 0.007505 | 0.673861 |
| GOTERM_BP_FAT | GO:0001819~positive regulation of cytokine production | 6 | 25 | 8.81 | 0.007505 | 0.673861 |
| GOTERM_BP_FAT | GO:0009607~response to biotic stimulus | 9 | 25 | 4.45 | 0.007505 | 0.673861 |
| GOTERM_BP_FAT | GO:0002376~immune system process | 11 | 25 | 3.43 | 0.007639 | 0.685896 |
| GOTERM_BP_FAT | GO:0048870~cell motility | 8 | 25 | 5.23 | 0.007730 | 0.694045 |
| GOTERM_BP_FAT | GO:0030155~regulation of cell adhesion | 7 | 25 | 6.48 | 0.007784 | 0.698860 |
| GOTERM_BP_FAT | GO:0007088~regulation of mitotic nuclear division | 4 | 25 | 25.16 | 0.008028 | 0.720804 |
| GOTERM_BP_FAT | GO:0007346~regulation of mitotic cell cycle | 6 | 25 | 8.52 | 0.008127 | 0.729697 |
| GOTERM_BP_FAT | GO:0032101~regulation of response to external stimulus | 8 | 25 | 5.15 | 0.008127 | 0.729697 |
| GOTERM_BP_FAT | GO:0006959~humoral immune response | 5 | 25 | 12.77 | 0.008249 | 0.740655 |
| GOTERM_MF_FAT | GO:0070412~R-SMAD binding | 3 | 23 | 85.75 | 0.019630 | 1.705575 |
| WIKIPATHWAYS | WP4891:COVID 19 adverse outcome pathway | 3 | 20 | 82.02 | 0.016587 | 1.491965 |
| GOTERM_BP_FAT | GO:0070848~response to growth factor | 6 | 25 | 8.11 | 0.009868 | 0.886047 |
| GOTERM_BP_FAT | GO:0042119~neutrophil activation | 3 | 25 | 77.36 | 0.010347 | 0.929060 |
| GOTERM_MF_FAT | GO:0000978~RNA polymerase II cis-regulatory region sequence-specific DNA binding | 8 | 23 | 4.81 | 0.019630 | 1.705575 |
| GOTERM_BP_FAT | GO:0051241~negative regulation of multicellular organismal process | 8 | 25 | 4.88 | 0.010729 | 0.963328 |
| GOTERM_MF_FAT | GO:0140677~molecular function activator activity | 8 | 23 | 4.80 | 0.019630 | 1.705575 |
| GOTERM_BP_FAT | GO:0044419~biological process involved in interspecies interaction between organisms | 9 | 25 | 4.11 | 0.011286 | 1.013343 |
| KEGG_PATHWAY | hsa05171:Coronavirus disease - COVID-19 | 5 | 17 | 10.96 | 0.007362 | 0.536099 |
| GOTERM_MF_FAT | GO:0000987~cis-regulatory region sequence-specific DNA binding | 8 | 23 | 4.71 | 0.019630 | 1.705575 |
| KEGG_PATHWAY | hsa04064:NF-kappa B signaling pathway | 4 | 17 | 19.87 | 0.007568 | 0.551093 |
| GOTERM_BP_FAT | GO:0051783~regulation of nuclear division | 4 | 25 | 20.49 | 0.013344 | 1.198123 |
| GOTERM_BP_FAT | GO:0007165~signal transduction | 15 | 25 | 2.29 | 0.013579 | 1.219191 |
| GOTERM_BP_FAT | GO:0043066~negative regulation of apoptotic process | 7 | 25 | 5.68 | 0.013632 | 1.223957 |
| GOTERM_BP_FAT | GO:0016477~cell migration | 7 | 25 | 5.68 | 0.013632 | 1.223957 |
| GOTERM_BP_FAT | GO:0032743~positive regulation of interleukin-2 production | 3 | 25 | 64.46 | 0.013849 | 1.243496 |
| GOTERM_BP_FAT | GO:0006935~chemotaxis | 5 | 25 | 10.80 | 0.013849 | 1.243496 |
| GOTERM_BP_FAT | GO:0042330~taxis | 5 | 25 | 10.74 | 0.013969 | 1.254197 |
| GOTERM_BP_FAT | GO:0051240~positive regulation of multicellular organismal process | 9 | 25 | 3.93 | 0.013969 | 1.254197 |
| WIKIPATHWAYS | WP2355:Corticotropin releasing hormone signaling | 4 | 20 | 19.02 | 0.026060 | 2.343984 |
| GOTERM_MF_FAT | GO:0030545~signaling receptor regulator activity | 6 | 23 | 7.20 | 0.022110 | 1.921006 |
| GOTERM_BP_FAT | GO:0002274~myeloid leukocyte activation | 4 | 25 | 19.10 | 0.015071 | 1.353167 |
| GOTERM_BP_FAT | GO:0045321~leukocyte activation | 6 | 25 | 7.16 | 0.015139 | 1.359245 |
| GOTERM_BP_FAT | GO:0043069~negative regulation of programmed cell death | 7 | 25 | 5.47 | 0.015511 | 1.392673 |
| GOTERM_BP_FAT | GO:0071495~cellular response to endogenous stimulus | 7 | 25 | 5.45 | 0.015607 | 1.401295 |
| GOTERM_BP_FAT | GO:0040011~locomotion | 5 | 25 | 10.13 | 0.016455 | 1.477425 |
| GOTERM_BP_FAT | GO:0042127~regulation of cell population proliferation | 9 | 25 | 3.79 | 0.016663 | 1.496132 |
| GOTERM_BP_FAT | GO:0048660~regulation of smooth muscle cell proliferation | 4 | 25 | 17.38 | 0.018669 | 1.676193 |
| GOTERM_BP_FAT | GO:0032675~regulation of interleukin-6 production | 4 | 25 | 17.29 | 0.018797 | 1.687752 |
| GOTERM_BP_FAT | GO:0051093~negative regulation of developmental process | 7 | 25 | 5.15 | 0.019941 | 1.790406 |
| GOTERM_BP_FAT | GO:0045429~positive regulation of nitric oxide biosynthetic process | 3 | 25 | 50.45 | 0.019941 | 1.790406 |
| GOTERM_MF_FAT | GO:0000981~DNA-binding transcription factor activity, RNA polymerase II-specific | 8 | 23 | 4.19 | 0.030247 | 2.628028 |
| GOTERM_BP_FAT | GO:0045840~positive regulation of mitotic nuclear division | 3 | 25 | 49.38 | 0.020443 | 1.835493 |
| GOTERM_BP_FAT | GO:1904407~positive regulation of nitric oxide metabolic process | 3 | 25 | 49.38 | 0.020443 | 1.835493 |
| GOTERM_MF_FAT | GO:0000977~RNA polymerase II transcription regulatory region sequence-specific DNA binding | 8 | 23 | 4.12 | 0.030247 | 2.628028 |
| KEGG_PATHWAY | hsa04010:MAPK signaling pathway | 5 | 17 | 8.69 | 0.013382 | 0.974454 |
| GOTERM_BP_FAT | GO:0023052~signaling | 15 | 25 | 2.15 | 0.022472 | 2.017653 |
| GOTERM_BP_FAT | GO:0030097~hemopoiesis | 6 | 25 | 6.39 | 0.022472 | 2.017708 |
| KEGG_PATHWAY | hsa05135:Yersinia infection | 4 | 17 | 15.12 | 0.013382 | 0.974454 |
| KEGG_PATHWAY | hsa05162:Measles | 4 | 17 | 15.01 | 0.013382 | 0.974454 |
| WIKIPATHWAYS | WP4659:Gastrin signaling | 4 | 20 | 15.22 | 0.042930 | 3.861400 |
| GOTERM_BP_FAT | GO:0007154~cell communication | 15 | 25 | 2.13 | 0.023961 | 2.151399 |
| GOTERM_BP_FAT | GO:0030334~regulation of cell migration | 7 | 25 | 4.91 | 0.023961 | 2.151399 |
| GOTERM_BP_FAT | GO:0051707~response to other organism | 8 | 25 | 4.07 | 0.024450 | 2.195287 |
| GOTERM_MF_FAT | GO:0008009~chemokine activity | 3 | 23 | 43.72 | 0.032193 | 2.797086 |
| GOTERM_BP_FAT | GO:0043207~response to external biotic stimulus | 8 | 25 | 4.06 | 0.024523 | 2.201788 |
| KEGG_PATHWAY | hsa04380:Osteoclast differentiation | 4 | 17 | 14.59 | 0.013548 | 0.986532 |
| GOTERM_BP_FAT | GO:0009617~response to bacterium | 6 | 25 | 6.12 | 0.026034 | 2.337484 |
| GOTERM_BP_FAT | GO:0019221~cytokine-mediated signaling pathway | 5 | 25 | 8.61 | 0.026087 | 2.342228 |
| GOTERM_MF_FAT | GO:0003700~DNA-binding transcription factor activity | 8 | 23 | 3.95 | 0.032193 | 2.797086 |
| GOTERM_BP_FAT | GO:0050727~regulation of inflammatory response | 5 | 25 | 8.54 | 0.026722 | 2.399228 |
| WIKIPATHWAYS | WP5088:Prostaglandin signaling | 3 | 20 | 39.77 | 0.042930 | 3.861400 |
| GOTERM_BP_FAT | GO:0009605~response to external stimulus | 9 | 25 | 3.39 | 0.030178 | 2.709534 |
| GOTERM_BP_FAT | GO:2000145~regulation of cell motility | 7 | 25 | 4.64 | 0.030381 | 2.727781 |
| GOTERM_MF_FAT | GO:0000976~transcription cis-regulatory region binding | 8 | 23 | 3.83 | 0.033195 | 2.884116 |
| GOTERM_MF_FAT | GO:0001067~transcription regulatory region nucleic acid binding | 8 | 23 | 3.83 | 0.033195 | 2.884116 |
| WIKIPATHWAYS | WP3617:Photodynamic therapy induced NF kB survival signaling | 3 | 20 | 37.50 | 0.042930 | 3.861400 |
| WIKIPATHWAYS | WP5095:Overview of proinflammatory and profibrotic mediators | 4 | 20 | 13.56 | 0.042930 | 3.861400 |
| GOTERM_BP_FAT | GO:0010564~regulation of cell cycle process | 6 | 25 | 5.84 | 0.030776 | 2.763273 |
| KEGG_PATHWAY | hsa04932:Non-alcoholic fatty liver disease | 4 | 17 | 13.29 | 0.016583 | 1.207501 |
| GOTERM_BP_FAT | GO:0009628~response to abiotic stimulus | 7 | 25 | 4.59 | 0.031460 | 2.824653 |
| GOTERM_BP_FAT | GO:1902895~positive regulation of miRNA transcription | 3 | 25 | 37.43 | 0.031460 | 2.824653 |
| WIKIPATHWAYS | WP366:TGF beta signaling pathway | 4 | 20 | 13.26 | 0.042930 | 3.861400 |
| GOTERM_BP_FAT | GO:1901654~response to ketone | 4 | 25 | 13.39 | 0.032969 | 2.960201 |
| GOTERM_BP_FAT | GO:0051785~positive regulation of nuclear division | 3 | 25 | 36.26 | 0.032969 | 2.960201 |
| GOTERM_BP_FAT | GO:0051247~positive regulation of protein metabolic process | 7 | 25 | 4.50 | 0.033625 | 3.019039 |
| WIKIPATHWAYS | WP4630:Measles virus infection | 4 | 20 | 12.87 | 0.043132 | 3.879606 |
| GOTERM_BP_FAT | GO:0040012~regulation of locomotion | 7 | 25 | 4.47 | 0.034640 | 3.110157 |
| GOTERM_BP_FAT | GO:0010629~negative regulation of gene expression | 8 | 25 | 3.73 | 0.035817 | 3.215882 |
| GOTERM_MF_FAT | GO:1990837~sequence-specific double-stranded DNA binding | 8 | 23 | 3.68 | 0.038887 | 3.378722 |
| GOTERM_BP_FAT | GO:0045428~regulation of nitric oxide biosynthetic process | 3 | 25 | 34.13 | 0.036025 | 3.234515 |
| GOTERM_BP_FAT | GO:0002685~regulation of leukocyte migration | 4 | 25 | 12.68 | 0.036902 | 3.313320 |
| GOTERM_BP_FAT | GO:0010638~positive regulation of organelle organization | 5 | 25 | 7.57 | 0.037292 | 3.348334 |
| GOTERM_BP_FAT | GO:0080164~regulation of nitric oxide metabolic process | 3 | 25 | 33.15 | 0.037292 | 3.348334 |
| GOTERM_BP_FAT | GO:2001234~negative regulation of apoptotic signaling pathway | 4 | 25 | 12.43 | 0.038213 | 3.431004 |
| GOTERM_MF_FAT | GO:0005125~cytokine activity | 4 | 23 | 12.33 | 0.038887 | 3.378722 |
| GOTERM_BP_FAT | GO:0045595~regulation of cell differentiation | 8 | 25 | 3.66 | 0.038297 | 3.438592 |
| GOTERM_BP_FAT | GO:0022407~regulation of cell-cell adhesion | 5 | 25 | 7.45 | 0.038319 | 3.440568 |
| GOTERM_MF_FAT | GO:0003677~DNA binding | 10 | 23 | 2.82 | 0.038887 | 3.378722 |
| GOTERM_BP_FAT | GO:2000630~positive regulation of miRNA metabolic process | 3 | 25 | 31.79 | 0.039333 | 3.531532 |
| GOTERM_BP_FAT | GO:0071409~cellular response to cycloheximide | 2 | 25 | 515.71 | 0.039507 | 3.547195 |
| GOTERM_BP_FAT | GO:0009725~response to hormone | 6 | 25 | 5.33 | 0.040190 | 3.608496 |
| GOTERM_BP_FAT | GO:0071363~cellular response to growth factor stimulus | 5 | 25 | 7.18 | 0.042634 | 3.827976 |
| KEGG_PATHWAY | hsa05134:Legionellosis | 3 | 17 | 27.94 | 0.026924 | 1.960460 |
| GOTERM_MF_FAT | GO:0042379~chemokine receptor binding | 3 | 23 | 28.58 | 0.040532 | 3.521646 |
| GOTERM_MF_FAT | GO:0003690~double-stranded DNA binding | 8 | 23 | 3.46 | 0.040532 | 3.521646 |
| GOTERM_BP_FAT | GO:0031347~regulation of defense response | 6 | 25 | 5.12 | 0.047116 | 4.230400 |
| GOTERM_MF_FAT | GO:0046332~SMAD binding | 3 | 23 | 28.22 | 0.040532 | 3.521646 |
| GOTERM_BP_FAT | GO:1902893~regulation of miRNA transcription | 3 | 25 | 28.30 | 0.047665 | 4.279704 |
| GOTERM_MF_FAT | GO:0043565~sequence-specific DNA binding | 8 | 23 | 3.44 | 0.040532 | 3.521646 |
| GOTERM_BP_FAT | GO:0090068~positive regulation of cell cycle process | 4 | 25 | 11.09 | 0.049293 | 4.425835 |
| KEGG_PATHWAY | hsa05167:Kaposi sarcoma-associated herpesvirus infection | 4 | 17 | 10.64 | 0.027578 | 2.008086 |
| GOTERM_MF_FAT | GO:0048018~receptor ligand activity | 5 | 23 | 6.68 | 0.042265 | 3.672238 |
| KEGG_PATHWAY | hsa05130:Pathogenic Escherichia coli infection | 4 | 17 | 10.28 | 0.028819 | 2.098442 |
| GOTERM_MF_FAT | GO:0030546~signaling receptor activator activity | 5 | 23 | 6.58 | 0.042789 | 3.717757 |
| GOTERM_MF_FAT | GO:0005126~cytokine receptor binding | 4 | 23 | 10.39 | 0.043007 | 3.736691 |
| KEGG_PATHWAY | hsa05031:Amphetamine addiction | 3 | 17 | 22.67 | 0.034390 | 2.504109 |
| KEGG_PATHWAY | hsa05120:Epithelial cell signaling in Helicobacter pylori infection | 3 | 17 | 22.03 | 0.034620 | 2.520869 |
| KEGG_PATHWAY | hsa05140:Leishmaniasis | 3 | 17 | 19.80 | 0.040629 | 2.958453 |
| KEGG_PATHWAY | hsa05132:Salmonella infection | 4 | 17 | 8.31 | 0.042801 | 3.116585 |
| KEGG_PATHWAY | hsa05210:Colorectal cancer | 3 | 17 | 17.98 | 0.044844 | 3.265323 |

**Supplemental Table 5**. Enrichment analysis of common DEGs in DC

| **Category** | **Term** | **Count** | **List Total** | **Fold Enrichment** | **Benjamini** | **FDR** |
| --- | --- | --- | --- | --- | --- | --- |
| GOTERM_BP_FAT | GO:0019219~regulation of nucleobase-containing compound metabolic process | 10 | 10 | 4.52 | 0.000934 | 0.086512 |
| GOTERM_MF_FAT | GO:0001228~DNA-binding transcription activator activity, RNA polymerase II-specific | 6 | 10 | 20.68 | 0.000137 | 0.012212 |
| GOTERM_MF_FAT | GO:0001216~DNA-binding transcription activator activity | 6 | 10 | 20.40 | 0.000137 | 0.012212 |
| GOTERM_BP_FAT | GO:0009719~response to endogenous stimulus | 7 | 10 | 10.48 | 0.002304 | 0.213340 |
| GOTERM_BP_FAT | GO:2001141~regulation of RNA biosynthetic process | 9 | 10 | 4.82 | 0.002719 | 0.251813 |
| GOTERM_BP_FAT | GO:0051252~regulation of RNA metabolic process | 9 | 10 | 4.39 | 0.003425 | 0.317149 |
| GOTERM_BP_FAT | GO:0010557~positive regulation of macromolecule biosynthetic process | 8 | 10 | 5.87 | 0.003425 | 0.317149 |
| GOTERM_BP_FAT | GO:1901700~response to oxygen-containing compound | 7 | 10 | 8.10 | 0.003425 | 0.317149 |
| GOTERM_BP_FAT | GO:0009891~positive regulation of biosynthetic process | 8 | 10 | 5.61 | 0.003445 | 0.319060 |
| GOTERM_BP_FAT | GO:0071495~cellular response to endogenous stimulus | 6 | 10 | 11.71 | 0.003445 | 0.319060 |
| WIKIPATHWAYS | WP2355:Corticotropin releasing hormone signaling | 4 | 9 | 42.67 | 0.006725 | 0.672465 |
| GOTERM_BP_FAT | GO:0006351~DNA-templated transcription | 5 | 10 | 16.96 | 0.006921 | 0.640958 |
| GOTERM_BP_FAT | GO:0045935~positive regulation of nucleobase-containing compound metabolic process | 7 | 10 | 6.40 | 0.007658 | 0.709153 |
| GOTERM_BP_FAT | GO:0045944~positive regulation of transcription by RNA polymerase II | 6 | 10 | 9.28 | 0.007658 | 0.709153 |
| GOTERM_BP_FAT | GO:0010604~positive regulation of macromolecule metabolic process | 8 | 10 | 4.57 | 0.007996 | 0.740486 |
| GOTERM_MF_FAT | GO:0000978~RNA polymerase II cis-regulatory region sequence-specific DNA binding | 6 | 10 | 8.48 | 0.005422 | 0.483604 |
| GOTERM_CC_FAT | GO:0000785~chromatin | 6 | 9 | 8.06 | 0.005401 | 0.540094 |
| GOTERM_BP_FAT | GO:0006355~regulation of DNA-templated transcription | 8 | 10 | 4.30 | 0.011052 | 1.023480 |
| GOTERM_MF_FAT | GO:0000987~cis-regulatory region sequence-specific DNA binding | 6 | 10 | 8.28 | 0.005422 | 0.483604 |
| GOTERM_BP_FAT | GO:0009893~positive regulation of metabolic process | 8 | 10 | 4.17 | 0.012584 | 1.165375 |
| GOTERM_BP_FAT | GO:0009968~negative regulation of signal transduction | 6 | 10 | 7.78 | 0.013003 | 1.204178 |
| GOTERM_BP_FAT | GO:0071345~cellular response to cytokine stimulus | 5 | 10 | 11.90 | 0.014302 | 1.324509 |
| GOTERM_MF_FAT | GO:0000981~DNA-binding transcription factor activity, RNA polymerase II-specific | 6 | 10 | 7.31 | 0.006590 | 0.587741 |
| GOTERM_BP_FAT | GO:0023057~negative regulation of signaling | 6 | 10 | 7.28 | 0.014302 | 1.324509 |
| GOTERM_BP_FAT | GO:0010648~negative regulation of cell communication | 6 | 10 | 7.28 | 0.014302 | 1.324509 |
| GOTERM_MF_FAT | GO:0000977~RNA polymerase II transcription regulatory region sequence-specific DNA binding | 6 | 10 | 7.20 | 0.006590 | 0.587741 |
| GOTERM_BP_FAT | GO:0010468~regulation of gene expression | 9 | 10 | 3.03 | 0.014302 | 1.324509 |
| GOTERM_BP_FAT | GO:0009725~response to hormone | 5 | 10 | 11.14 | 0.014302 | 1.324509 |
| GOTERM_BP_FAT | GO:0010556~regulation of macromolecule biosynthetic process | 9 | 10 | 2.98 | 0.014302 | 1.324509 |
| GOTERM_BP_FAT | GO:0045893~positive regulation of DNA-templated transcription | 6 | 10 | 6.88 | 0.014302 | 1.324509 |
| GOTERM_BP_FAT | GO:1902680~positive regulation of RNA biosynthetic process | 6 | 10 | 6.88 | 0.014302 | 1.324509 |
| GOTERM_MF_FAT | GO:0003700~DNA-binding transcription factor activity | 6 | 10 | 6.85 | 0.006590 | 0.587741 |
| GOTERM_BP_FAT | GO:0006357~regulation of transcription by RNA polymerase II | 7 | 10 | 4.91 | 0.014302 | 1.324509 |
| GOTERM_BP_FAT | GO:0033993~response to lipid | 5 | 10 | 10.78 | 0.014302 | 1.324509 |
| GOTERM_BP_FAT | GO:0034097~response to cytokine | 5 | 10 | 10.59 | 0.014708 | 1.362090 |
| GOTERM_MF_FAT | GO:0000976~transcription cis-regulatory region binding | 6 | 10 | 6.69 | 0.006590 | 0.587741 |
| GOTERM_MF_FAT | GO:0001067~transcription regulatory region nucleic acid binding | 6 | 10 | 6.69 | 0.006590 | 0.587741 |
| GOTERM_BP_FAT | GO:1901652~response to peptide | 5 | 10 | 10.41 | 0.015129 | 1.401092 |
| GOTERM_MF_FAT | GO:1990837~sequence-specific double-stranded DNA binding | 6 | 10 | 6.41 | 0.007220 | 0.643950 |
| GOTERM_BP_FAT | GO:0048585~negative regulation of response to stimulus | 6 | 10 | 6.17 | 0.020676 | 1.914792 |
| GOTERM_BP_FAT | GO:0051254~positive regulation of RNA metabolic process | 6 | 10 | 6.08 | 0.020947 | 1.939839 |
| GOTERM_BP_FAT | GO:0006366~transcription by RNA polymerase II | 4 | 10 | 17.88 | 0.020947 | 1.939839 |
| GOTERM_MF_FAT | GO:0003690~double-stranded DNA binding | 6 | 10 | 6.03 | 0.008331 | 0.743004 |
| GOTERM_MF_FAT | GO:0043565~sequence-specific DNA binding | 6 | 10 | 5.98 | 0.008331 | 0.743004 |
| GOTERM_BP_FAT | GO:0009892~negative regulation of metabolic process | 7 | 10 | 3.99 | 0.035512 | 3.288702 |
| GOTERM_BP_FAT | GO:0007166~cell surface receptor signaling pathway | 6 | 10 | 5.33 | 0.035512 | 3.288702 |
| GOTERM_BP_FAT | GO:0071363~cellular response to growth factor stimulus | 4 | 10 | 14.34 | 0.036019 | 3.335617 |
| GOTERM_BP_FAT | GO:0051384~response to glucocorticoid | 3 | 10 | 41.57 | 0.039399 | 3.648650 |
| GOTERM_MF_FAT | GO:0140110~transcription regulator activity | 6 | 10 | 5.10 | 0.015997 | 1.426753 |
| GOTERM_BP_FAT | GO:0070848~response to growth factor | 4 | 10 | 13.52 | 0.039733 | 3.679552 |
| GOTERM_BP_FAT | GO:0032870~cellular response to hormone stimulus | 4 | 10 | 13.45 | 0.039733 | 3.679552 |
| GOTERM_BP_FAT | GO:0031960~response to corticosteroid | 3 | 10 | 36.15 | 0.047684 | 4.415862 |

**Supplemental Table 6**. Enrichment analysis of common DEGs in NK cells

| **Category** | **Term** | **Count** | **List Total** | **Fold Enrichment** | **Benjamini** | **FDR** |
| --- | --- | --- | --- | --- | --- | --- |
| GOTERM_CC_FAT | GO:0035976~transcription factor AP-1 complex | 3 | 6 | 1474.75 | 1.92E-05 | 0.001915 |
| GOTERM_MF_FAT | GO:0001228~DNA-binding transcription activator activity, RNA polymerase II-specific | 5 | 7 | 24.62 | 0.000492 | 0.041295 |
| GOTERM_MF_FAT | GO:0001216~DNA-binding transcription activator activity | 5 | 7 | 24.28 | 0.000492 | 0.041295 |
| GOTERM_BP_FAT | GO:0140467~integrated stress response signaling | 3 | 7 | 230.96 | 0.025041 | 2.394092 |
| WIKIPATHWAYS | WP366:TGF beta signaling pathway | 4 | 7 | 38.23 | 0.007579 | 0.562124 |
| GOTERM_CC_FAT | GO:0000785~chromatin | 5 | 6 | 10.07 | 0.001637 | 0.163662 |
| WIKIPATHWAYS | WP4877:Host pathogen interaction of human coronaviruses MAPK signaling | 3 | 7 | 105.14 | 0.009664 | 0.716748 |
| GOTERM_CC_FAT | GO:0005667~transcription regulator complex | 4 | 6 | 22.56 | 0.001637 | 0.163662 |
| GOTERM_MF_FAT | GO:0000978~RNA polymerase II cis-regulatory region sequence-specific DNA binding | 5 | 7 | 10.10 | 0.008511 | 0.713784 |
| GOTERM_MF_FAT | GO:0000987~cis-regulatory region sequence-specific DNA binding | 5 | 7 | 9.85 | 0.008511 | 0.713784 |
| WIKIPATHWAYS | WP5115:Network map of SARS CoV 2 signaling | 4 | 7 | 19.87 | 0.009664 | 0.716748 |
| WIKIPATHWAYS | WP286:IL3 signaling | 3 | 7 | 77.25 | 0.009664 | 0.716748 |
| WIKIPATHWAYS | WP5373:Osteoarthritic chondrocyte hypertrophy | 3 | 7 | 75.70 | 0.009664 | 0.716748 |
| WIKIPATHWAYS | WP3611:Photodynamic therapy induced AP 1 survival signaling | 3 | 7 | 74.22 | 0.009664 | 0.716748 |
| GOTERM_MF_FAT | GO:0000981~DNA-binding transcription factor activity, RNA polymerase II-specific | 5 | 7 | 8.70 | 0.008622 | 0.723172 |
| GOTERM_MF_FAT | GO:0000977~RNA polymerase II transcription regulatory region sequence-specific DNA binding | 5 | 7 | 8.58 | 0.008622 | 0.723172 |
| GOTERM_MF_FAT | GO:0003700~DNA-binding transcription factor activity | 5 | 7 | 8.16 | 0.008622 | 0.723172 |
| GOTERM_MF_FAT | GO:0000976~transcription cis-regulatory region binding | 5 | 7 | 7.97 | 0.008622 | 0.723172 |
| GOTERM_MF_FAT | GO:0001067~transcription regulatory region nucleic acid binding | 5 | 7 | 7.96 | 0.008622 | 0.723172 |
| GOTERM_MF_FAT | GO:1990837~sequence-specific double-stranded DNA binding | 5 | 7 | 7.63 | 0.009122 | 0.765093 |
| KEGG_PATHWAY | hsa04668:TNF signaling pathway | 3 | 5 | 43.03 | 0.035457 | 3.491111 |
| GOTERM_MF_FAT | GO:0003690~double-stranded DNA binding | 5 | 7 | 7.18 | 0.009938 | 0.833549 |
| GOTERM_MF_FAT | GO:0043565~sequence-specific DNA binding | 5 | 7 | 7.12 | 0.009938 | 0.833549 |
| WIKIPATHWAYS | WP2355:Corticotropin releasing hormone signaling | 3 | 7 | 41.14 | 0.026860 | 1.992108 |
| KEGG_PATHWAY | hsa05418:Fluid shear stress and atherosclerosis | 3 | 5 | 36.31 | 0.035457 | 3.491111 |
| KEGG_PATHWAY | hsa04380:Osteoclast differentiation | 3 | 5 | 35.81 | 0.035457 | 3.491111 |
| GOTERM_CC_FAT | GO:0090575~RNA polymerase II transcription regulator complex | 3 | 6 | 34.56 | 0.010127 | 1.012656 |
| WIKIPATHWAYS | WP5353:Macrophage stimulating protein MSP signaling | 3 | 7 | 35.05 | 0.032284 | 2.394367 |
| GOTERM_MF_FAT | GO:0140110~transcription regulator activity | 5 | 7 | 6.07 | 0.016814 | 1.410243 |
| GOTERM_MF_FAT | GO:0061629~RNA polymerase II-specific DNA-binding transcription factor binding | 3 | 7 | 20.40 | 0.040260 | 3.376666 |
| GOTERM_MF_FAT | GO:0003677~DNA binding | 5 | 7 | 4.64 | 0.040260 | 3.376666 |

**Supplemental Table 7**. Enrichment analysis of common DEGs in CD4+ T Cells

| **Category** | **Term** | **Count** | **List Total** | **Fold Enrichment** | **Benjamini** | **FDR** |
| --- | --- | --- | --- | --- | --- | --- |
| GOTERM_CC_FAT | GO:0035976~transcription factor AP-1 complex | 4 | 12 | 983.17 | 3.0E-07 | 0.000029 |
| GOTERM_BP_FAT | GO:0071277~cellular response to calcium ion | 5 | 13 | 95.67 | 9.7E-05 | 0.009189 |
| WIKIPATHWAYS | WP5373:Osteoarthritic chondrocyte hypertrophy | 5 | 11 | 80.29 | 2.5E-05 | 0.002253 |
| GOTERM_BP_FAT | GO:0048545~response to steroid hormone | 6 | 13 | 30.35 | 2.4E-04 | 0.022439 |
| GOTERM_BP_FAT | GO:0051592~response to calcium ion | 5 | 13 | 53.68 | 3.3E-04 | 0.031337 |
| GOTERM_BP_FAT | GO:0009719~response to endogenous stimulus | 8 | 13 | 9.21 | 5.0E-04 | 0.046883 |
| GOTERM_BP_FAT | GO:0006357~regulation of transcription by RNA polymerase II | 10 | 13 | 5.39 | 5.0E-04 | 0.046883 |
| GOTERM_BP_FAT | GO:0071248~cellular response to metal ion | 5 | 13 | 40.78 | 5.0E-04 | 0.046883 |
| REACTOME_PATHWAY | R-HSA-9031628~NGF-stimulated transcription | 4 | 11 | 107.17 | 7.9E-04 | 0.075467 |
| GOTERM_BP_FAT | GO:0009725~response to hormone | 7 | 13 | 11.99 | 7.0E-04 | 0.066251 |
| WIKIPATHWAYS | WP366:TGF beta signaling pathway | 5 | 11 | 30.41 | 6.2E-04 | 0.056499 |
| KEGG_PATHWAY | hsa04380:Osteoclast differentiation | 5 | 11 | 27.13 | 1.1E-03 | 0.099825 |
| REACTOME_PATHWAY | R-HSA-198725~Nuclear Events (kinase and transcription factor activation) | 4 | 11 | 68.52 | 1.5E-03 | 0.147107 |
| GOTERM_BP_FAT | GO:0006355~regulation of DNA-templated transcription | 10 | 13 | 4.14 | 2.9E-03 | 0.273252 |
| GOTERM_BP_FAT | GO:2001141~regulation of RNA biosynthetic process | 10 | 13 | 4.12 | 2.9E-03 | 0.273252 |
| GOTERM_BP_FAT | GO:0010557~positive regulation of macromolecule biosynthetic process | 9 | 13 | 5.08 | 2.9E-03 | 0.273252 |
| GOTERM_BP_FAT | GO:0051254~positive regulation of RNA metabolic process | 8 | 13 | 6.23 | 2.9E-03 | 0.275402 |
| GOTERM_BP_FAT | GO:0010038~response to metal ion | 5 | 13 | 21.26 | 2.9E-03 | 0.275402 |
| GOTERM_BP_FAT | GO:0045944~positive regulation of transcription by RNA polymerase II | 7 | 13 | 8.33 | 2.9E-03 | 0.275402 |
| GOTERM_BP_FAT | GO:0009891~positive regulation of biosynthetic process | 9 | 13 | 4.85 | 2.9E-03 | 0.275402 |
| GOTERM_BP_FAT | GO:0070887~cellular response to chemical stimulus | 8 | 13 | 5.78 | 3.9E-03 | 0.371872 |
| GOTERM_BP_FAT | GO:0051252~regulation of RNA metabolic process | 10 | 13 | 3.75 | 3.9E-03 | 0.371872 |
| GOTERM_BP_FAT | GO:0045935~positive regulation of nucleobase-containing compound metabolic process | 8 | 13 | 5.62 | 4.4E-03 | 0.414175 |
| REACTOME_PATHWAY | R-HSA-187037~Signaling by NTRK1 (TRKA) | 4 | 11 | 36.66 | 6.7E-03 | 0.639525 |
| WIKIPATHWAYS | WP2355:Corticotropin releasing hormone signaling | 4 | 11 | 34.91 | 5.5E-03 | 0.502164 |
| GOTERM_BP_FAT | GO:0033993~response to lipid | 6 | 13 | 9.95 | 5.9E-03 | 0.559899 |
| GOTERM_BP_FAT | GO:0019219~regulation of nucleobase-containing compound metabolic process | 10 | 13 | 3.48 | 6.3E-03 | 0.589814 |
| KEGG_PATHWAY | hsa04657:IL-17 signaling pathway | 4 | 11 | 32.67 | 5.7E-03 | 0.515270 |
| GOTERM_BP_FAT | GO:0007565~female pregnancy | 4 | 13 | 32.62 | 7.0E-03 | 0.660900 |
| REACTOME_PATHWAY | R-HSA-166520~Signaling by NTRKs | 4 | 11 | 31.43 | 7.7E-03 | 0.735361 |
| GOTERM_BP_FAT | GO:0000122~negative regulation of transcription by RNA polymerase II | 6 | 13 | 9.29 | 7.0E-03 | 0.660900 |
| GOTERM_BP_FAT | GO:0010468~regulation of gene expression | 11 | 13 | 2.85 | 7.0E-03 | 0.660900 |
| REACTOME_PATHWAY | R-HSA-162582~Signal Transduction | 9 | 11 | 3.58 | 7.7E-03 | 0.735361 |
| GOTERM_BP_FAT | GO:0140467~integrated stress response signaling | 3 | 13 | 124.37 | 7.4E-03 | 0.698590 |
| GOTERM_BP_FAT | GO:0010604~positive regulation of macromolecule metabolic process | 9 | 13 | 3.96 | 7.4E-03 | 0.698590 |
| GOTERM_BP_FAT | GO:0010556~regulation of macromolecule biosynthetic process | 11 | 13 | 2.80 | 7.4E-03 | 0.698590 |
| GOTERM_BP_FAT | GO:0044703~multi-organism reproductive process | 4 | 13 | 29.26 | 7.5E-03 | 0.711314 |
| REACTOME_PATHWAY | R-HSA-9018519~Estrogen-dependent gene expression | 4 | 11 | 27.68 | 7.7E-03 | 0.735361 |
| GOTERM_BP_FAT | GO:0045893~positive regulation of DNA-templated transcription | 7 | 13 | 6.18 | 7.6E-03 | 0.716056 |
| GOTERM_BP_FAT | GO:1902680~positive regulation of RNA biosynthetic process | 7 | 13 | 6.17 | 7.6E-03 | 0.716056 |
| GOTERM_BP_FAT | GO:0044706~multi-multicellular organism process | 4 | 13 | 28.03 | 7.7E-03 | 0.724088 |
| GOTERM_MF_FAT | GO:0001228~DNA-binding transcription activator activity, RNA polymerase II-specific | 5 | 13 | 13.26 | 2.1E-02 | 1.911569 |
| GOTERM_MF_FAT | GO:0001216~DNA-binding transcription activator activity | 5 | 13 | 13.08 | 2.1E-02 | 1.911569 |
| GOTERM_BP_FAT | GO:0070848~response to growth factor | 5 | 13 | 13.00 | 8.6E-03 | 0.814753 |
| REACTOME_PATHWAY | R-HSA-2262752~Cellular responses to stress | 6 | 11 | 7.76 | 8.4E-03 | 0.801903 |
| GOTERM_BP_FAT | GO:0009612~response to mechanical stimulus | 4 | 13 | 25.62 | 9.4E-03 | 0.881559 |
| GOTERM_BP_FAT | GO:0051726~regulation of cell cycle | 6 | 13 | 7.90 | 9.8E-03 | 0.928132 |
| GOTERM_BP_FAT | GO:0009893~positive regulation of metabolic process | 9 | 13 | 3.61 | 1.1E-02 | 1.019910 |
| GOTERM_CC_FAT | GO:0090575~RNA polymerase II transcription regulator complex | 4 | 12 | 23.04 | 1.9E-02 | 1.812017 |
| REACTOME_PATHWAY | R-HSA-8953897~Cellular responses to stimuli | 6 | 11 | 6.82 | 1.3E-02 | 1.281768 |
| WIKIPATHWAYS | WP437:EGF EGFR signaling | 4 | 11 | 20.20 | 1.9E-02 | 1.723482 |
| WIKIPATHWAYS | WP4877:Host pathogen interaction of human coronaviruses MAPK signaling | 3 | 11 | 66.91 | 1.9E-02 | 1.723482 |
| GOTERM_BP_FAT | GO:0045892~negative regulation of DNA-templated transcription | 6 | 13 | 6.85 | 1.8E-02 | 1.697503 |
| GOTERM_BP_FAT | GO:1902679~negative regulation of RNA biosynthetic process | 6 | 13 | 6.79 | 1.8E-02 | 1.707719 |
| REACTOME_PATHWAY | R-HSA-8939211~ESR-mediated signaling | 4 | 11 | 18.74 | 1.6E-02 | 1.537495 |
| GOTERM_BP_FAT | GO:0030099~myeloid cell differentiation | 4 | 13 | 18.95 | 1.9E-02 | 1.828882 |
| GOTERM_BP_FAT | GO:0051253~negative regulation of RNA metabolic process | 6 | 13 | 6.29 | 2.4E-02 | 2.293893 |
| WIKIPATHWAYS | WP2873:Aryl hydrocarbon receptor pathway | 3 | 11 | 53.53 | 2.4E-02 | 2.160325 |
| GOTERM_CC_FAT | GO:0000785~chromatin | 6 | 12 | 6.04 | 3.2E-02 | 3.101556 |
| GOTERM_MF_FAT | GO:0140110~transcription regulator activity | 7 | 13 | 4.58 | 4.8E-02 | 4.342077 |
| WIKIPATHWAYS | WP286:IL3 signaling | 3 | 11 | 49.16 | 2.4E-02 | 2.160325 |
| GOTERM_MF_FAT | GO:0046332~SMAD binding | 3 | 13 | 49.43 | 4.8E-02 | 4.342077 |
| WIKIPATHWAYS | WP3611:Photodynamic therapy induced AP 1 survival signaling | 3 | 11 | 47.23 | 2.4E-02 | 2.160325 |
| GOTERM_BP_FAT | GO:0045934~negative regulation of nucleobase-containing compound metabolic process | 6 | 13 | 5.75 | 3.6E-02 | 3.349328 |
| WIKIPATHWAYS | WP2374:Oncostatin M signaling | 3 | 11 | 43.01 | 2.5E-02 | 2.312408 |
| REACTOME_PATHWAY | R-HSA-9006931~Signaling by Nuclear Receptors | 4 | 11 | 14.07 | 3.3E-02 | 3.169890 |
| GOTERM_BP_FAT | GO:0007179~transforming growth factor beta receptor signaling pathway | 3 | 13 | 43.05 | 3.9E-02 | 3.659959 |
| WIKIPATHWAYS | WP382:MAPK signaling | 4 | 11 | 13.00 | 2.9E-02 | 2.670864 |
| GOTERM_BP_FAT | GO:1903706~regulation of hemopoiesis | 4 | 13 | 13.32 | 4.8E-02 | 4.532406 |
| WIKIPATHWAYS | WP5115:Network map of SARS CoV 2 signaling | 4 | 11 | 12.64 | 2.9E-02 | 2.670864 |
| GOTERM_BP_FAT | GO:0071495~cellular response to endogenous stimulus | 5 | 13 | 7.51 | 5.0E-02 | 4.673355 |
| GOTERM_BP_FAT | GO:0033554~cellular response to stress | 6 | 13 | 5.22 | 5.0E-02 | 4.688245 |
| WIKIPATHWAYS | WP2840:Hair follicle development cytodifferentiation stage 3 of 3 | 3 | 11 | 27.69 | 4.5E-02 | 4.127226 |
| WIKIPATHWAYS | WP2882:Nuclear receptors meta pathway | 4 | 11 | 10.23 | 4.5E-02 | 4.127226 |

**Supplemental Table 8**. Enrichment analysis of common DEGs in CD8+ T cells

| **Category** | **Term** | **Count** | **List Total** | **Fold Enrichment** | **Benjamini** | **FDR** |
| --- | --- | --- | --- | --- | --- | --- |
| GOTERM_CC_FAT | GO:0035976~transcription factor AP-1 complex | 3 | 8 | 1106.06 | 0.000157 | 0.015680 |
| GOTERM_BP_FAT | GO:0071277~cellular response to calcium ion | 4 | 9 | 110.55 | 0.002208 | 0.219795 |
| GOTERM_BP_FAT | GO:0051592~response to calcium ion | 4 | 9 | 62.03 | 0.006282 | 0.625267 |
| GOTERM_BP_FAT | GO:0071248~cellular response to metal ion | 4 | 9 | 47.12 | 0.009526 | 0.948091 |
| GOTERM_BP_FAT | GO:0048545~response to steroid hormone | 4 | 9 | 29.23 | 0.029466 | 2.932784 |
| REACTOME_PATHWAY | R-HSA-162582~Signal Transduction | 8 | 9 | 3.89 | 0.025583 | 2.527336 |
| GOTERM_BP_FAT | GO:0010038~response to metal ion | 4 | 9 | 24.57 | 0.039340 | 3.915516 |
| REACTOME_PATHWAY | R-HSA-9031628~NGF-stimulated transcription | 3 | 9 | 98.24 | 0.025583 | 2.527336 |
| WIKIPATHWAYS | WP5373:Osteoarthritic chondrocyte hypertrophy | 3 | 8 | 66.24 | 0.035345 | 3.371984 |
| REACTOME_PATHWAY | R-HSA-198725~Nuclear Events (kinase and transcription factor activation) | 3 | 9 | 62.81 | 0.041800 | 4.129287 |
| WIKIPATHWAYS | WP2374:Oncostatin M signaling | 3 | 8 | 59.14 | 0.035345 | 3.371984 |

**Supplemental Table 9**. Enrichment analysis of common DEGs in B Cells

| **Category** | **Term** | **Count** | **List Total** | **Fold Enrichment** | **Benjamini** | **FDR** |
| --- | --- | --- | --- | --- | --- | --- |
| GOTERM_BP_FAT | GO:0006357~regulation of transcription by RNA polymerase II | 7 | 7 | 7.01 | 0.004381 | 0.414663 |
| GOTERM_BP_FAT | GO:0006355~regulation of DNA-templated transcription | 7 | 7 | 5.38 | 0.006429 | 0.608455 |
| GOTERM_BP_FAT | GO:2001141~regulation of RNA biosynthetic process | 7 | 7 | 5.36 | 0.006429 | 0.608455 |
| GOTERM_BP_FAT | GO:0033993~response to lipid | 5 | 7 | 15.40 | 0.006429 | 0.608455 |
| GOTERM_BP_FAT | GO:0048545~response to steroid hormone | 4 | 7 | 37.58 | 0.006429 | 0.608455 |
| GOTERM_BP_FAT | GO:0051252~regulation of RNA metabolic process | 7 | 7 | 4.88 | 0.006429 | 0.608455 |
| GOTERM_BP_FAT | GO:0019219~regulation of nucleobase-containing compound metabolic process | 7 | 7 | 4.52 | 0.00871 | 0.824371 |
| REACTOME_PATHWAY | R-HSA-9031628~NGF-stimulated transcription | 3 | 7 | 126.31 | 0.012179 | 1.151204 |
| GOTERM_BP_FAT | GO:0071277~cellular response to calcium ion | 3 | 7 | 106.60 | 0.015485 | 1.465632 |
| REACTOME_PATHWAY | R-HSA-198725~Nuclear Events (kinase and transcription factor activation) | 3 | 7 | 80.75 | 0.014963 | 1.414276 |
| WIKIPATHWAYS | WP5373:Osteoarthritic chondrocyte hypertrophy | 3 | 7 | 75.70 | 0.033901 | 3.064989 |
| GOTERM_BP_FAT | GO:0010468~regulation of gene expression | 7 | 7 | 3.37 | 0.033342 | 3.155654 |
| GOTERM_BP_FAT | GO:0051592~response to calcium ion | 3 | 7 | 59.82 | 0.033342 | 3.155654 |
| GOTERM_BP_FAT | GO:0051384~response to glucocorticoid | 3 | 7 | 59.39 | 0.033342 | 3.155654 |
| GOTERM_BP_FAT | GO:0010556~regulation of macromolecule biosynthetic process | 7 | 7 | 3.31 | 0.033342 | 3.155654 |
| GOTERM_BP_FAT | GO:0031960~response to corticosteroid | 3 | 7 | 51.64 | 0.03983 | 3.769788 |
| GOTERM_BP_FAT | GO:0009888~tissue development | 5 | 7 | 7.41 | 0.03983 | 3.769788 |
| GOTERM_BP_FAT | GO:0051254~positive regulation of RNA metabolic process | 5 | 7 | 7.24 | 0.03983 | 3.769788 |
| GOTERM_BP_FAT | GO:0071248~cellular response to metal ion | 3 | 7 | 45.44 | 0.03983 | 3.769788 |
| GOTERM_BP_FAT | GO:0007565~female pregnancy | 3 | 7 | 45.44 | 0.03983 | 3.769788 |
| REACTOME_PATHWAY | R-HSA-187037~Signaling by NTRK1 (TRKA) | 3 | 7 | 43.21 | 0.034679 | 3.277833 |
| GOTERM_BP_FAT | GO:0044703~multi-organism reproductive process | 3 | 7 | 40.76 | 0.044516 | 4.213246 |
| GOTERM_BP_FAT | GO:0009725~response to hormone | 4 | 7 | 12.73 | 0.044516 | 4.213246 |
| GOTERM_CC_FAT | GO:0035976~transcription factor AP-1 complex | 2 | 6 | 983.17 | 0.045745 | 4.574461 |
| GOTERM_BP_FAT | GO:0044706~multi-multicellular organism process | 3 | 7 | 39.04 | 0.044516 | 4.213246 |
| GOTERM_BP_FAT | GO:0045935~positive regulation of nucleobase-containing compound metabolic process | 5 | 7 | 6.53 | 0.044516 | 4.213246 |
| REACTOME_PATHWAY | R-HSA-166520~Signaling by NTRKs | 3 | 7 | 37.04 | 0.035289 | 3.335547 |
| GOTERM_BP_FAT | GO:0009612~response to mechanical stimulus | 3 | 7 | 35.69 | 0.049609 | 4.695331 |
| PIR_SUPERFAMILY | PIRSF000939:MAPK_Ptase | 2 | 2 | 277.29 | 0.003606 | 0.360639 |
